# Dissimilar gene repertoires of *Dickeya solani* involved in the colonization of lesions and roots of *Solanum tuberosum*

**DOI:** 10.3389/fpls.2023.1154110

**Published:** 2023-05-08

**Authors:** Kévin Robic, Euphrasie Munier, Géraldine Effantin, Joy Lachat, Delphine Naquin, Erwan Gueguen, Denis Faure

**Affiliations:** ^1^ French Federation of Seed Potato Growers (FN3PT/inov3PT), Paris, France; ^2^ Université Paris-Saclay, CEA, CNRS, Institute for Integrative Biology of the Cell (I2BC), Gif-sur-Yvette, France; ^3^ Univ Lyon, Université Claude Bernard Lyon1, CNRS, INSA Lyon, UMR5240 MAP, Lyon, France

**Keywords:** blackleg disease, Tn-seq, soft rot, *Solanum tuberosrum*, root colonization

## Abstract

*Dickeya* and *Pectobacterium* species are necrotrophic pathogens that macerate stems (blackleg disease) and tubers (soft rot disease) of *Solanum tuberosum*. They proliferate by exploiting plant cell remains. They also colonize roots, even if no symptoms are observed. The genes involved in pre-symptomatic root colonization are poorly understood. Here, transposon-sequencing (Tn-seq) analysis of *Dickeya solani* living in macerated tissues revealed 126 genes important for competitive colonization of tuber lesions and 207 for stem lesions, including 96 genes common to both conditions. Common genes included *acr* genes involved in the detoxification of plant defense phytoalexins and *kduD, kduI, eda* (*=kdgA*)*, gudD, garK, garL*, and *garR* genes involved in the assimilation of pectin and galactarate. In root colonization, Tn-seq highlighted 83 genes, all different from those in stem and tuber lesion conditions. They encode the exploitation of organic and mineral nutrients (*dpp*, *ddp*, *dctA*, and *pst*) including glucuronate (*kdgK* and *yeiQ*) and synthesis of metabolites: cellulose (*celY* and *bcs*), aryl polyene (*ape*), and oocydin (*ooc*). We constructed in-frame deletion mutants of *bcsA, ddpA, apeH*, and *pstA* genes. All mutants were virulent in stem infection assays, but they were impaired in the competitive colonization of roots. In addition, the Δ*pstA* mutant was impaired in its capacity to colonize progeny tubers. Overall, this work distinguished two metabolic networks supporting either an oligotrophic lifestyle on roots or a copiotrophic lifestyle in lesions. This work revealed novel traits and pathways important for understanding how the *D. solani* pathogen efficiently survives on roots, persists in the environment, and colonizes progeny tubers.

## Introduction

*Dickeya solani* emerged in potato tuber cultivation (*Solanum tuberosum*) in Europe at the beginning of the 2000s (van der Wolf et al., 2014). A retrospective analysis of potato pathogen archives revealed that some *D. solani* had been isolated on rare occasions in Switzerland in the 1990s (Pédron et al., 2021). *D. solani* as well as other species of *Dickeya* and *Pectobacterium* genera (order of *Enterobacterales*) are the causative agents of soft rot disease on tubers and blackleg disease on stems of potato plants (van der Wolf et al., 2021). They also cause damage to a wide range of plants of agronomical interest worldwide (van der Wolf et al., 2021). The necrotrophic pathogens *Dickeya* and *Pectobacterium* secrete plant cell wall macerating enzymes as their main virulence factors and then proliferate in lesions (macerated tissues) by exploiting plant cell remains as nutrients (Van Gijsegem et al., 2021). How these pathogens persist and disseminate in agrosystems (soil, surface waters, plant reservoirs, insect vectors, and propagation *via* progeny tubers) and, more specifically, how they survive by colonizing and exploiting roots of their host (underground lifestyle) remain questions under investigation.

Comparative genomics has shown little genetic variation in *D. solani* isolates collected in Europe consistent with a bottleneck associated with a recent spread in potato and bulb plants (van der Wolf et al., 2014; Khayi et al., 2015; Golanowska et al., 2018; Blin P. et al., 2021). The primary host(s) of *D. solani* remain(s) uncertain. Horizontal gene transfer events have been observed in a few *D. solani* isolates, replacing some *D. solani* genes by their orthologous counterparts acquired from *Dickeya dianthicola* (Khayi et al., 2015). These transfer events suggest ecological promiscuity between the two species. Variation in the degree of symptoms caused to the potato host was also observed among *D. solani* isolates: these phenotypes were tentatively associated with single-nucleotide polymorphism in some genes (*fliC*, *fliN*, *fhaB2*, and *vfmB*) and the presence or absence of some other loci (*arcZ*, *mtgA*, and *hrpQ*) (Khayi et al., 2015; Golanowska et al., 2018; Blin P. et al., 2021). The involvement of these genetic variations in virulence remains to be confirmed by reverse genetics. Several studies compared *D. solani*, *D. dadantii*, and *D. dianthicola*, highlighting some distinctive traits in *D. solani* (Potrykus et al., 2015; Potrykus et al., 2018; Raoul des Essarts et al., 2019). Notably, some specific regulatory sequences trigger a higher expression of *pelD* and *pelE* genes, encoding macerating enzymes, in *D. solani* compared to *D. dianthicola* (Blin P. et al., 2021; Duprey et al., 2016). High expression of *pelD* and *pelE* would contribute to the capacity of *D. solani* to cause rotting at a lower bacterial inoculum than *D. dianthicola* (Blin P. et al., 2021; Raoul des Essarts et al., 2019). A comparative analysis of metabolic capabilities showed that *D. solani* exploited a wider range of compounds as nitrogen source than *D. dianthicola* (Raoul des Essarts et al., 2019). Transcriptomics of the pathogens recovered from tuber lesions pinpointed the higher expression of the glyoxylate shunt in *D. solani* than in *D. dianthicola*, a metabolic pathway that contributes to the exploitation of alternative sources of carbon when sugar is limiting (Raoul des Essarts et al., 2019). All these traits could facilitate the settlement of *D. solani* in the potato plant host.

The invasion success of *D. solani* could also be related to its capacity to survive under stressful conditions and to compete with the microbiota of the potato host. The *D. solani* genome contains several polyketide synthase (PKS) and non-ribosomal peptide synthetase (NRPS) clusters coding for different secondary metabolites: aryl-polyene, oocydin, zeamine, and solanimycin. Aryl-polyene synthesis is encoded by the *ape* genes that are widespread in β-proteobacteria and γ-proteobacteria, including several *Enterobacterales* such as *D. solani* and some strains of *Escherichia coli* (Schöner et al., 2016; Johnston et al., 2021). Aryl-polyenes are pigments anchored to the outer membrane of bacterial cells that may contribute to oxidative stress response and biofilm formation (Johnston et al., 2021). A recent study (Brual et al., 2021) highlighted three other PKS/NRPKS clusters coding for synthesis of oocydin, zeamine, and solanimycin, which are present in all published genomes of *D. solani*, but not all three together in the other *Dickeya* species. The *ooc* cluster is highly similar to the *ooc* genes coding for synthesis of oocydin A, a chlorinated macrolide present in some *Serratia* and *Dickeya* strains (Matilla et al., 2012). In different *Dickeya* and *Serratia* strains, the *zms* cluster is responsible for synthesis of zeamine, a cationic polyamine-polyketide (Zhou et al., 2011). The *sol* cluster is present in different *Dickeya* species and encodes solanimycin that is active against a broad range of micro-eukaryotes (Brual et al., 2021; Matilla et al., 2022). As compared to a wild-type strain of *D. solani* Ds0432-1, the constructed mutants Δ*solG*, Δ*zmsA*, and Δ*oocL* were impaired in antibiosis against yeasts, Gram-positive bacteria, and Ascomyceta, respectively (Brual et al., 2021). The contribution of these secondary metabolites to plant colonization by *D. solani* is not known.

*D. solani* colonizes stems, xylem vessels, and leaves, as well as roots and stolons from which develop progeny tubers (Czajkowski et al., 2010). Under greenhouse conditions, when inoculated into pots, *D. solani* rapidly colonizes the roots (2 weeks post-inoculation of soil) and, to a lesser extent, stems even if no symptoms (blackleg lesions) were observed (Czajkowski et al., 2010). Several weeks later, blackleg symptoms develop in stems with an incidence mainly depending on pathogen abundance in the inoculum and wounding of roots; root wounding facilitates entry of pathogens (Czajkowski et al., 2010; Blin P. et al., 2021). At the end of the vegetative cycle, pathogens may cause soft rot in progeny tubers or may colonize them without symptoms. Transcriptomics revealed differentially expressed genes in *D. solani* living in soft rot lesions in potato tubers (Raoul des Essarts et al., 2019). In contrast, the lifestyle of *D. solani* and other pectinolytic *Dickeya* and *Pectobacterium* when they colonize roots remains poorly investigated. In a recent study, 10,000 Tn*5*-mutants carrying a promoterless *gusA* gene were screened to identify *D. solani* genes differentially expressed in the presence of leaves, stems, roots, and tubers of *S. tuberosum* (Czajkowski et al., 2020). A single gene (*pstB*) was identified as expressed in the presence of either root or tuber tissues, but not stem or leaf (Czajkowski et al., 2020). The *pstABC* genes encode a transporter involved in importing phosphate and phosphate-mediated gene regulation, including pathogeny, in different *Enterobacterales* (Daigle et al., 1995; Runyen-Janecky et al., 2005; Röder et al., 2021). Whether *pst* genes contribute to colonization and survival of *D. solani* on roots is not known.

Transposon-sequencing (Tn-seq) identifies insertional mutations that confer an advantage or a defect under a given condition. In several plant pathogens, Tn-seq unveiled genes involved in the competitive colonization of lesions caused by necrotrophic and biotrophic pathogens (Duong et al., 2018; Helmann et al., 2019; Royet et al., 2019; Su et al., 2021; Torres et al., 2021; Helmann et al., 2022; Luneau et al., 2022; Morinière et al., 2022). Tn-seq analyses revealed a large set of genes associated with the colonization of lesions provoked by *D. dadantii* on chicory leaves and by *D. dianthicola* and *D. dadantii* on potato tubers (Royet et al., 2019; Helmann et al., 2022). Tn-seq also permitted discovery of genes associated with the competitive colonization of roots in pathogenic and beneficial bacteria, under gnotobiotic conditions (Cole et al., 2017; Liu et al., 2018; Sivakumar et al., 2019). To our knowledge, a unique Tn-seq study explored the root colonization of a plant pathogen under non-gnotobiotic conditions using a non-sterile substrate for plant growth (Torres et al., 2021). Identification of genes associated with an underground lifestyle of pathogens could be useful for preventing disease by targeting root colonization traits of pathogens.

In this work, we constructed a Tn-mutant library in the necrotroph *D. solani* RNS 08.23.3.1.A (=PRI3337) to identify genes involved in the colonization of *S. tuberosum* roots under a non-gnotobiotic condition. We also searched for genes associated with the colonization of the lesions of stems (blackleg disease) and tubers (soft rot disease). In addition, we identified the *D. solani* genes contributing to growth on different nutrients derived from plant cell wall—pectin, galacturonate, glucoronate, and galactarate—and compared them with those identified in lesions. Then, we focused on four genes *bscA* (cellulose synthesis), *dppA* (peptide transport), *pstA* (phosphate importation), and *apeH* (aryl polyene synthesis) that we identified by Tn-seq as important for the competitive colonization of roots. The *dppA*, *pstA*, *bcsA*, and *apeH* genes had been characterized in some *Enterobacteriales*, especially in *E. coli* and *D. dadantii* (Olson et al., 1991; Daigle et al., 1995; Mee-Ngan et al., 2005; Runyen-Janecky et al., 2005; Prigent-Combaret et al., 2012; Schöner et al., 2016; Johnston et al., 2021; Röder et al., 2021), but their role in root colonization is not known. We constructed in-frame deletions of these genes in *D. solani*, and we validated their implication in the underground lifestyle of *D. solani*. We showed that the *pstA* gene is also important for colonizing progeny tubers. Overall, this work highlights a novel landscape of genes and traits involved in *D. solani–S. tuberosum* interactions, especially those involved in the underground lifestyle of this plant pathogen.

## Materials and methods

### Bacteria and plant material

Bacterial strains are described in **Table 1** and culture conditions are discussed in the **Supplementary Materials SI1**. Tubers (G0 generation, caliber 35–45 mm) of *S. tuberosum* variety Bintje were provided by *Comité Nord Plants de Pomme de Terre* (CNPPT, Achicourt, France).

**Table 1:** Bacterial strains and plasmids.

Bacterial strains and plasmids	Characteristics	Reference
RNS 08.23.3.1A	*Dickeya solani* wild type	Khayi et al., 2018
RNS 08.23.3.1A Rif^R^	RNS 08.23.3.1A stain with spontaneous mutation of the *rpoB* gene conferring resistance to Rif	This study
RNS 08.23.3.1A Rif^R^-Gm^R^	RNS 08.23.3.1A Rif^R^ strain containing a Gm^R^ cassette at the Tn7 insertion site	This study
Δ*bcsA*	Deletion of gene *bcsA* (DS0823_v1_2374) in the RNS 08.23.3.1A Rif^R^-Gm^R^ strain	This study
Δ*dppA*	Deletion of gene *dppA* (DS0823_v1_2392) in the RNS 08.23.3.1A Rif^R^ Gm^R^ strain	This study
Δ*pstA*	Deletion of gene *pstA* (DS0823_v1_2527) in the RNS 08.23.3.1A Rif^R^ Gm^R^ strain	This study
Δ*apeH*	Deletion of gene *apeH* (DS0823_v1_2513) in the RNS 08.23.3.1A Rif^R^ Gm^R^ strain	This study
*Escherichia coli* MFD*pir*	*RP4-2-Tc::(ΔMu1::aac(3)IV-ΔaphA-Δnic35-ΔMu2::zeo) ΔdapA::erm-pir) ΔrecA*	Jackson et al., 2020
*Escherichia coli* DH5λ*pir*	λpir phage lysogen of DH5α	Laboratory collection
pSAM-Ec	Suicide mobilizable vector; Amp^R^, Km^R^; Km-resistance gene bordered by mariner inverted repeat sequence containing MmeI restriction site; himar1-C9 transposase gene under the control of P*lac.*	Wiles et al., 2013
pRE112	Suicide vector for allelic exchange in *D. solani*; Cm^R^, *sacB*, *oriT* RP4	Edwards et al., 1998
pMobile-CRISPRi_1	Plasmid encoding mobilizable transposase Tn7	Peters et al., 2019
pTn7-M	Suicide vector for Gm-resistance gene insertion at Tn7 site; Km^R^, Gm^R^, *oriR6K*, *Tn7L* and *Tn7R* extremities, multiple cloning site, *oriT* RP4	Zobel et al., 2015

### Construction of transposon library in *Dickeya solani* RNS 08.23.3.1.A

The genome of *D. solani* RNS 08.23.3.1A contains a circular chromosome of 4,922,468 bp in which 169,132 TA dinucleotides are all potential sites for the insertion of the *himar1* mariner transposon (**Figure S1**). Among them, 132,173 TA sites are distributed in 99.5% of the 4,518 coding sequences. Only 22 genes do not contain any TA dinucleotide in their sequence and could not be considered in this study (listed in **Table S1**).

A library of Tn-mutants was constructed in a rifampicin-resistant (Rif^R^) derivative of *D. solani* RNS 08.23.3.1A using the plasmid pSAM-Ec, which harbors a modified *Himar1* mariner associated to a kanamycin (Km) resistance cassette (Wiles et al., 2013). Five hundred conjugation matings between *E. coli* MFDpir carrying pSAM-Ec and *D. solani* RNS 08.23.3.1A Rif^R^ were performed as described by Gonzalez-Mula et al. (2019) (experimental details in SI2). Approximately 1.8 × 10^7^ independent Tn-mutant colonies were recovered on agar TY medium supplemented with Rif and Km. This corresponds to approximately 100 times the number of TA sites present in the genome of *D. solani* RNS 08.23.3.1A (169,132 TA sites). All the Tn-mutant colonies were collected, and the resulting Tn-mutant library was homogenized, aliquoted, and stored in 25% glycerol at −80°C. Each aliquot contained 2.3 × 10^10^ CFU (colony-forming units) of Tn-mutants.

### Culture and analysis of the Tn-mutant library under different conditions

An aliquot of the Tn-mutant library was thawed on ice and then incubated at 28°C for 4 h in 10 ml of TY rich medium in order to reactivate the bacterial cells. Library cells were centrifugated and washed with a solution at 0.8% NaCl, and used for inoculating M9 minimal medium and plant materials. Four revived aliquots in TY rich medium were used to analyze the quality of the Tn-mutant library: these four replicates correspond to the TY rich medium condition. In the *in vitro* culture tests, the Tn-mutant library was diluted to obtain a final OD_600nm_ of 0.02 in 50 ml of liquid M9 medium. M9 medium was supplemented with different carbon sources at 2 g/L: sucrose (CAS 57-50-1), galacturonate (CAS 91510-62-2), glucuronate (CAS 6556-12-3), and galactarate (CAS 526-99-8), which were purchased from Sigma-Aldrich (Saint-Quentin-Fallavier, France), and pectin Dipecta AG366 (Agdia EMEA, Soisy sur Seine, France). This pectin is routinely used in crystal violet pectate medium for isolating *Dickeya* and *Pectobacterium* from environmental samples (Hélias et al., 2012). Cultures were incubated at 28°C for 24 h under shaking (200 rpm). Bacterial cells were centrifuged (4,000 rpm, 4°C, 15 min) and stored at −20°C before DNA extraction. Each culture condition was performed in four replicates.

In the *in planta* assays, the Tn-mutant library was inoculated in three conditions: roots and stems of entire plants and potato tubers. Potato plants were cultivated individually in 2-L pots containing horticultural compost (Floragard, Oldenburg, Germany) under non-gnotobiotic conditions in a greenhouse (16-h days and 8-h nights at 24°C). In the root colonization assay, 1 ml at 6 units OD_600nm_ of the Tn-mutant library was inoculated by watering (5.5 × 10^9^ CFU per pot). Forty plants were inoculated when the aerial part reached approximately 30 cm in height (4 weeks after tuber planting). In the stem infection assay, 50 µl at 1 unit OD_600nm_ of the Tn-mutant library was injected into the stem in the axil of the fourth leaf (5 × 10^7^ CFU per stem). These plants were developed from axillary buds of tubers and 140 stems were inoculated when they reached a size of approximately 20–30 cm in height. In the tuber infection assay, 10 µl at 1 unit OD_600nm_ of the Tn-mutant library was injected into the potato tubers (10^7^ CFU per tuber). Forty tubers were inoculated and then placed at 24°C in the dark. Plant and tuber assays were described previously (Blin P. et al., 2021).

Six weeks post-inoculation, the 40 root systems were collected and grouped into eight lots that we designated as the eight replicates of the root condition. Symptoms of maceration were not observed on root systems. Roots were crushed in 25 ml of a 0.8% NaCl solution during 1 min using a laboratory blender (Waring, Stamford, USA). The homogenate was filtered through miracloth (Millipore, Bedford, MA, USA) and then plated onto TY agar medium supplemented with Rif, Km, and cycloheximide. After 48 h at 28°C, the Tn-mutant colonies were collected and resuspended in 0.8% NaCl solution. For each replicate, 2 ml of bacterial suspension was pelleted and frozen prior to DNA extraction.

Five days post-inoculation, macerated tissues of the stems and tubers were collected and grouped into eight stem replicates (each from 15 stems) and four tuber replicates (each from 10 tubers). Each replicate was crushed in 10 ml of 0.8% NaCl solution and the Tn-mutant colonies were recovered using TY agar medium supplemented with Rif, Km, and cycloheximide, following the same protocol as for the root condition. For each replicate, 2 ml of bacterial suspension was centrifugated and frozen prior to DNA extraction.

### Analysis of the Tn-mutant library

Overall, 42 samples were analyzed. Four replicates were collected in the TY rich medium condition, in which the Tn-library was constructed. Four replicates were collected in M9 medium for each of the five carbon sources: sucrose, pectin, galacturonate, glucuronate, and galactarate. In addition, four replicates were collected in the macerated tuber condition, eight in the macerated stem condition, and eight in the root condition.

Total DNA from each replicate was extracted and then the Tn insertion sites were amplified (Primers in **Table S3**) and sequenced as described previously (Gonzalez-Mula et al., 2019; Torres et al., 2021). Illumina NextSeq 500 instrument (Illumina, San Diego, USA) was used in a single read 75 run at the I2BC-sequencing platform (I2BC, Gif-sur-Yvette, France). Each population generated over 3 million reads after specific cleaning of the Tn-seq data: transposon trimming with Trimmomatic (Bolger et al., 2014) and then barcode removal.

The reads were mapped using Bowtie-1.1.2 (Langmead et al., 2009) to the *D. solani* RNS 08.23.3.1A genome (Khayi et al., 2018; GenBank CP016928.1). Genome annotation was generated at the LABGeM (CEA/Genoscope CNRS UMR8030, Evry, France). Correspondence between the Genoscope annotation and NCBI annotation (released in April 2022 https://www.ncbi.nlm.nih.gov/data-hub/genome/GCA_000511285.2/) is provided in **Table S1**.

The mapping results (.bam files) were analyzed by the ARTIST pipeline (Pritchard et al., 2014) using MATLAB software (MathWorks, Natick, USA). All.bam files of the 42 compared conditions were deposited as sequence read archives (SRA) in the bioproject PRJNA939571 at the National Center for Biotechnology Information (**Table S4**). When a high correlation (*r*² ≥ 0.80) was observed in distribution and relative abundance of Tn insertions between the replicates of the same culture medium condition, the sequencing data were pooled for further analyses using ARTIST. Two ARTIST analyses were performed with a *p*-value cutoff of *p* < 0.01 in the Mann–Whitney *U* test: EL-ARTIST and Con-ARTIST (Pritchard et al., 2014). By comparing the number of Tn sequences at each AT insertion site in a given culture condition, the EL-ARTIST pipeline predicts the genes that are “non-essential” because they still contain Tn insertions (class 1), “essential” (class 2) because they contain no Tn insertions, or an “essential domain” (class 3) because no Tn insertions are present in a part of the gene. The “essential” term is used in the ARTIST suite to indicate a decrease (*p* < 0.01) in the relative abundance of Tn-mutants of a considered gene in a given condition. The Con-ARTIST pipeline compares two conditions, a test condition versus a reference condition. By comparing the number of Tn sequences at each AT insertion site in the reference and test conditions, Con-ARTIST distinguishes the genes with a conditionally “essential domain” (class 1), conditionally “essential genes” (class 2), genes with a conditionally “enriched domain” (class 3), conditionally “enriched genes” (class 4), and genes conditionally “non-essential, not enriched” (class 5). In this work, the Con-ARTIST class 2 “essential genes” were retained as candidate genes that are important for growth and survival in a given growth condition as compared to a reference condition. In the case of *in planta* culture conditions (roots and macerated stems and tubers), each replicate was analyzed separately and compared to the reference condition (TY rich medium) to provide a list of fitness genes (class 2 “essential genes”). Then, those that had been assigned to Con-ARTIST class 2 “essential genes” in at least half of the replicates of a considered condition were retained in a final unique list of fitness genes, as described by Torres et al. (2021).

The identified genes were categorized using COG classification (Clusters of Orthologous Groups of proteins) by Galperin et al. (2015). Antismash was used for identifying secondary metabolite biosynthetic gene clusters (https://antismash.secondarymetabolites.org; Blin K. et al., 2021). Some genes were manually analyzed, such as those associated with carbon metabolism according to the published data in *Enterobacteriales* (Tervo and Reed, 2012; Bouvier et al., 2019; Kuivanen et al., 2019).

### Construction of deletion mutants in *D. solani* by reverse genetics

In-frame deletions were constructed in four genes for which Tn-seq analysis predicted a contribution to competitive fitness in root colonization: *bcsA* (=DS0823_v1_2374) involved in cellulose synthesis, *dppA* (=DS0823_v1_2392) involved in peptide transport, *pstA* (=DS0823_v1_2527) involved in phosphate transport, and *apeH* (=DS0823_v1_2513) involved in synthesis of an unknown aryl-polyene compound. The mutated genes were chosen within a cluster coding for the same function; therefore, the same behavior was expected, even in the case of a potential polar effect.

The *D. solani* RNS 08.23.3.1A Rif^R^-Gm^R^ strain was obtained by integration of a Gm-resistance cassette at the unique *att*Tn*7* site of the *D. solani* genome, as described in SI3. The defective alleles were introduced in *D. solani* RNS 08.23.3.1A Rif^R^-Gm^R^ by conjugation (Edwards et al., 1998; Brual et al., 2021). A detailed protocol is presented in SI4. To validate the *in planta* behavior of the mutants, two independent clones were retained per genotype and further analyzed in plant assays.

### *In planta* behavior of the constructed *D. solani* deletion mutants

To measure the relative fitness of the four constructed deletion mutants (Rif^R^ Gm^R^), root colonization assays were performed in the presence of the ancestral strain *D. solani* RNS 08.23.3.1A Rif^R^. Competition between the ancestor *D. solani* RNS 08.23.3.1A Rif^R^ and its derivative *D. solani* RNS 08.23.3.1A Rif^R^-Gm^R^ was used as control to evaluate the potential bias caused by the Gm^R^ cassette.

The *D. solani* RNS 08.23.3.1A Rif^R^ strain and its derivatives were grown overnight in TY medium. The cells were rinsed twice in a 0.8% NaCl solution and then their OD_600nm_ was adjusted to 1 unit (10^9^ CFU/ml). Each Rif^R^ Gm^R^ strain was mixed with the Rif^R^ strain at a 1:1 ratio. For each mutant construction, two independent clones were analyzed. Each clone was inoculated in 10 pots. Six weeks post-inoculation, roots of each pot were sampled and 10 g was crushed in 30 ml of 0.8% NaCl solution following the same procedure as described above for the Tn-infection assays. The homogenate was diluted and spread on TY Rif and TY Rif-Gm agar medium, both being supplemented with cycloheximide, to enumerate the total *Dickeya* population and the mutant population. For each replicate, a competitive index (CI) was calculated (Macho et al., 2010) following the formula presented in SI5. A CI value equal to one indicated an equal fitness between *D. solani* RNS 08.23.3.1A Rif^R^ and *D. solani* RNS 08.23.3.1A Rif^R^-Gm^R^ (mutant strains), CI values greater than one indicated a fitness advantage of the RNS 08.23.3.1A Rif^R^-Gm^R^ mutant strain, and CI values below one indicated a fitness advantage of RNS 08.23.3.1A Rif^R^.

In addition to competition assays, each bacterial genotype was inoculated separately: in the first assay, pathogens were injected directly in stems, while in the second assay, pathogens were inoculated as described for the root colonization assay. For each mutant, two independent clones were analyzed. Each clone was inoculated on 10 plants. The strain RNS 08.23.3.1A Rif^R^-Gm^R^ was used as a virulent reference and uninoculated plants were used as negative control. In the stem inoculation assay, the length of internal lesions was measured 5 days post-infection. Abundance of pathogens was quantified in lesions (CFU/g of stem fresh weight) using numerations on TY supplemented with Rif, Gm, and cycloheximide. In the root inoculation assay, 8 weeks post-infection, abundance of pathogens in pots was quantified by counting on TY agar medium supplemented with Rif, Gm, and cycloheximide. The number of asymptomatic and symptomatic progeny tubers was counted, and finally, pathogen acquisition in tuber progeny was evaluated by testing asymptomatic progeny tubers for the presence of *D. solani* using qPCR (Blin P. et al., 2021).

### Statistics

The correlation between the different replicates of the Tn-seq mapping results was analyzed by calculating the linear regression coefficient (*r* ^2^). The traits measured in plants inoculated by the different bacterial genotypes (the CI values, length of the stem symptoms in cm, abundance of pathogens in rotten tissue or pots in CFU/g, and the number of symptomatic or asymptomatic plants) were compared by Kruskal–Wallis tests (*α* = 0.05). Then, a Dunn’s test was performed to compare traits of each constructed mutant with the strain RNS 08.23.3.1A Rif^R^-Gm^R^. For all statistical analyses and coefficient calculation, we used GraphPad Prism version 9.3.0, GraphPad Software (www.graphpad.com).

## Results

### Characteristics of the Tn-library in *D. solani* RNS 08.23.3.1A

**Figure 1:**
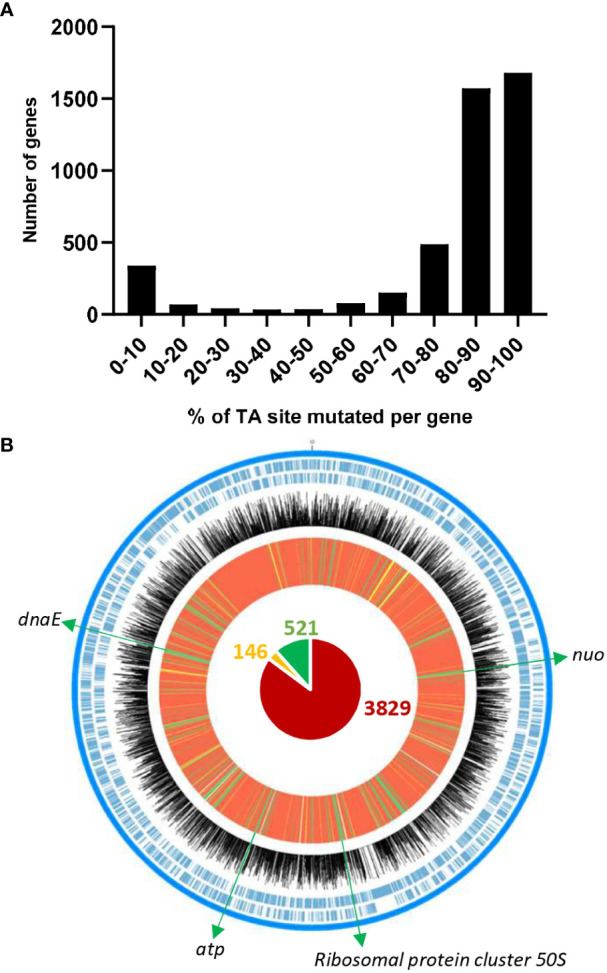
Characteristics of the Tn-library in *Dickeya solani*. The position and abundance of Tn insertions in the circular chromosome of *D. solani* RNS 08.23.3.1A Rif^R^ under TY rich medium conditions are shown. **(A)** Number of genes according to percentage of mutated TA sites per gene. **(B)** From the outside to the inside, the tracks represent the following: forward and reverse coding sequences in blue color; Log_10_ of number of Tn insertions per TA site for each gene in black; EL-ARTIST analysis that classified genes in three categories: “non-essential genes” in red, “genes with an essential domain” in yellow, and “essential genes” in green. In the center, the circle chart shows the total number of “non-essential genes” (3,829), “essential genes” (521), and “genes with an essential domain” (146). Examples of “essential genes” are indicated.

Using the *himar1* mariner transposon (Tn), we constructed a library of Tn-mutants in a rifampicin-resistant derivative of *D. solani* RNS 08.23.3.1A (**Table 1**). To examine the distribution of Tn in the *D. solani* genome, we extracted total DNA from four Tn-library aliquots that were grown in TY rich medium. By comparing the relative abundance of the Tn insertions along the circular chromosome, a high correlation (*r*² > 0.98) was observed between the four replicates. Sequencing data from the replicates were pooled for further analysis. In the *D. solani* Tn-library, more than 70% of genes have at least 80% of their TA sites with a Tn insertion (**Figure 1**), revealing good coverage of the genome. In the next step, the EL-ARTIST analysis (Pritchard et al., 2014) pinpointed 521 genes (“essential genes” according to the EL-ARTIST classification) for which Tn-mutants had impaired growth in TY medium. These 521 genes represented 11.5% of the total genes of *D. solani* RNS 08.23.3.1A. A similar percentage had been observed in previous Tn-seq studies (Hooven et al., 2016; Gonzalez-Mula et al., 2019; Royet et al., 2019). According to the COG classification, these 521 genes are associated with four main functional categories: bacterial metabolism (31.9%), information storage and processing (29.4%), cellular processes and signaling (25.3%), and poorly characterized genes (13.4%) (**Table S2**). Most of them encompass genes encoding for cell processes that are essential for bacterial viability and optimal growth: for instance, the *nuo* cluster (aerobic respiration), *atp* cluster (synthesis of ATP), *rpl* genes (50S ribosomal protein), and *dnaE* (DNA polymerase) (**Figure 1**). The Tn-library was thereafter challenged to several growth conditions to unveil and compare the different gene repertoires supporting the *D. solani* lifestyles.

### Fitness genes associated with growth of *D. solani* on plant cell wall components

Because *D. solani* secretes plant cell wall macerating enzymes, we searched for genes associated with growth of *D. solani* on nutrients derived from plant cell walls. The Tn-library was cultivated in M9 synthetic medium supplemented with different carbon sources: galacturonate, glucuronate, galactarate, and pectin. M9 medium with sucrose was used as a reference condition in comparative analyses. On average, 3 million reads were obtained and analyzed per replicate to determine the relative abundance of the Tn along the chromosome. We observed a good correlation between the four replicates of each growth condition: sucrose (*r* ^2^ > 0.94), galacturonate (*r* ^2^ > 0.98), glucuronate (*r* ^2^ > 0.88), galactarate (*r* ^2^ > 0.97), and pectin (*r* ^2^ > 0.99). Hence, we pooled sequencing data for each carbon source for further comparisons.

**Figure 2:**
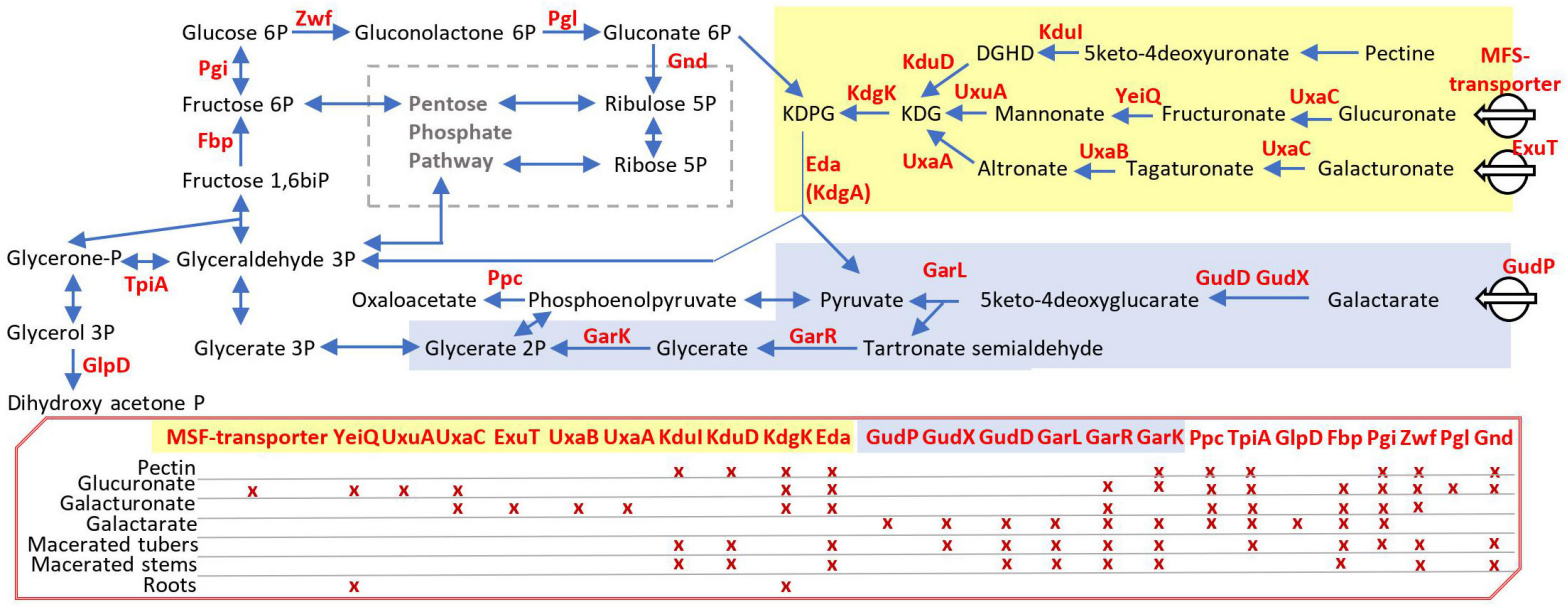
*Dickeya solani* pathways associated with competitive growth on plant cell wall components. This scheme exemplifies fitness genes pinpointed by Tn-seq (a complete list is available in **Table S2**) when the *D. solani* Tn-library was grown in M9 minimal medium with distinctive plant cell wall components as carbon and energy sources. In the schematic pathways, enzymes encoded by the identified genes are indicated in red; in the red box, crosses indicate for each enzyme in which growth condition they were identified using Tn-seq: pectin, glucuronate, galacturonate, and galactarate in M9 minimal medium, as well as *in planta* conditions: roots and rotted tubers and stems. DGHD, 3-deoxy-D-glycero-hexo-2,5-diulosonate; KDPG, 2-keto-3-deoxy-6-phosphogluconate; KDG, 2-keto-3-deoxy-gluconate.

Using EL-ARTIST, we analyzed the sequencing data obtained under sucrose growth conditions and then we used Con-ARTIST to compare the distribution of the Tn insertions in sucrose versus other conditions (**Table S2**). We focused on the “conditionally essential genes” (class 2) according to the Con-ARTIST analysis and we retained them as fitness genes. We identified 98 genes important for growth on pectin, 89 for growth on galacturonate, 96 for growth on glucoronate, and 87 for growth on galactarate (**Table S2**). Our Tn-seq experiments revealed some common and some nutrient-specific genes, which are important for the competitive growth of *D. solani* in the presence of plant cell wall components (**Figure 2**). Some common genes are involved in the pentose phosphate pathway and upper glycolysis/gluconeogenesis (*tpiA, fbp, pgi, zwf, pgl*, and *gnd*). Some nutrient-specific genes are associated with the importation and degradation of distinctive plant compounds: pectin (*kduI* and *kduD*), glucuronate (one unnamed MSF-transporter = DS0823_v1_1165, *yeiQ*, and *uxuA*), galacturonate (*exuT, uxaB*, and *uxaA*), and galactarate (*gudP, gudD, gudX*, and *garL*). The genes *kdgK* and *eda* (=*kdgA*) were important for fitness in growth on pectin, glucuronate, and galacturonate, but not galactarate. These genes reflect the metabolic pattern of *D. solani* in each of the tested growth conditions. Their identification was useful to interpret the fitness genes in complex environments such as roots and macerated stems and tubers.

### Fitness genes associated with the colonization of macerated stems, tubers, and roots

**Figure 3:**
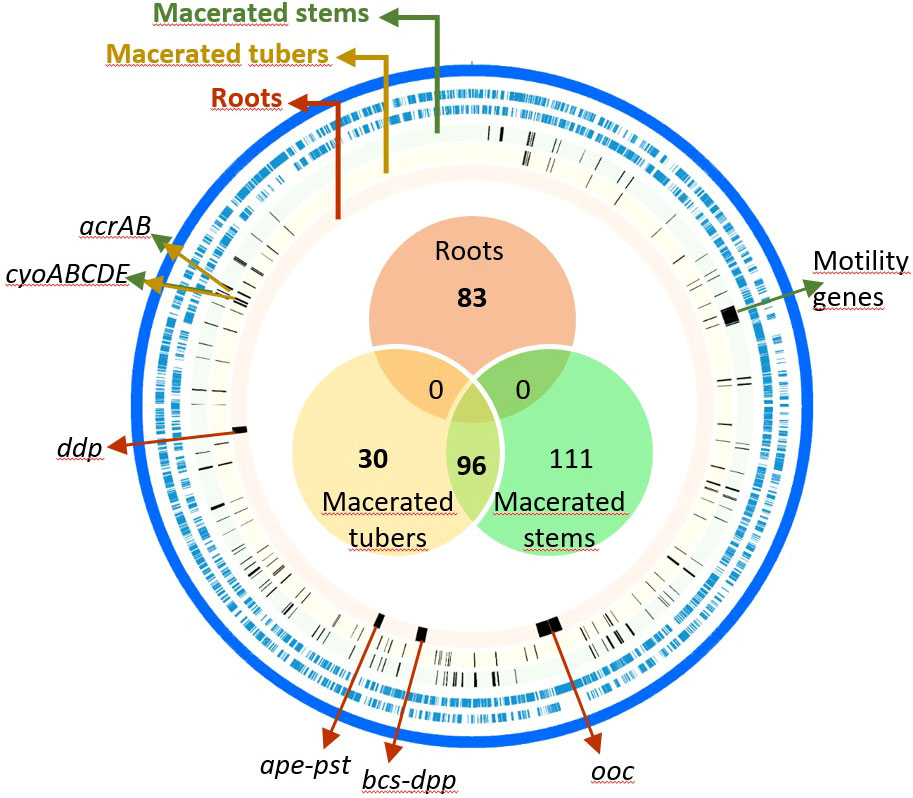
Genome map of *Dickeya solani* genes associated with competitive growth on roots and lesions in stems and tubers of *Solanum tuberosum*. From the outside to the inside, the tracks represent the following: forward and reverse coding sequence (in blue) and fitness genes (complete lists available in **Table S2**) when *D. solani* grew in macerated stems (green circle), in macerated tubers (yellow circle), and on roots (orange circle). The Venn diagram represents a comparison of fitness genes between the three *in planta* conditions. Some examples of fitness genes are indicated, such as a large cluster of motility genes that are important under macerated stem conditions, *arcAB* and *cyoABCDE* genes under macerated stem and tuber conditions, and the four clusters associated to fitness in roots condition, including *ddp*, *ape*, *pst*, *bcs*, *dpp*, and *ooc* genes.

To identify the *D. solani* genes involved in proliferation in macerated plant tissues, we inoculated 10^7^ CFU of the Tn-library on 40 tubers and 120 stems. After a 5-day incubation, *D. solani* reached 2.3 × 10^10^ ± 3.6 × 10^10^ CFU/g of lesions in tubers and 2.0 × 10^9^ ± 5.5 × 10^8^ CFU/g of lesions in stems. The macerated tubers and stems were assembled in four lots (each from 10 tubers) and eight lots (each from 15 stems), respectively. Each lot of macerated tissue is considered as a replicate. After DNA extraction and sequencing of Tn insertion sites, Con-ARTIST analysis was performed using TY medium as a reference condition. We identified 126 fitness genes important for the competitive colonization of tuber lesions and 207 for stem lesions, including 96 genes common to both conditions (**Table S2**). Most of these 237 genes are involved in metabolism (40%) and cellular processes and signaling (32%) categories according to the COG classification (**Table S2**). These genes are scattered along the *D. solani* genome (**Figure 3**). A noticeable exception is a large cluster of 39 genes coding for motility (*fli*, *flg*, and *flh*) and chemotaxis (*che* and *chp*), which were important for competitive fitness in stem lesions (**Figure 3** and **Table S2**). Additional operons associated with fitness in stem lesions are involved in the synthesis of purines (*purCDEHLM*) and different amino acids such arginine (*argABCEG*), histidine (*hisABCDFGHI*), leucine (*leuABCD*), valine, and isoleucine (*ilvCYDEM*). Some remarkable genes were important for fitness in both conditions, tuber and stem lesions: this category includes genes involved in upper glycolysis/gluconeogenesis (*fbp, zwf*, and *gnd*) and assimilation of pectin (*kduI, kduD*, and *eda*) or galactarate (*gudD, garL, garR*, and *garK*) (**Table S2**). Other common genes between macerated tuber and stem conditions were associated with detoxification (*acrAB* genes coding for an efflux system) and aerobic respiration (*cyoABCD* encoding the cytochrome o ubiquinol oxidase).

**Figure 4:**
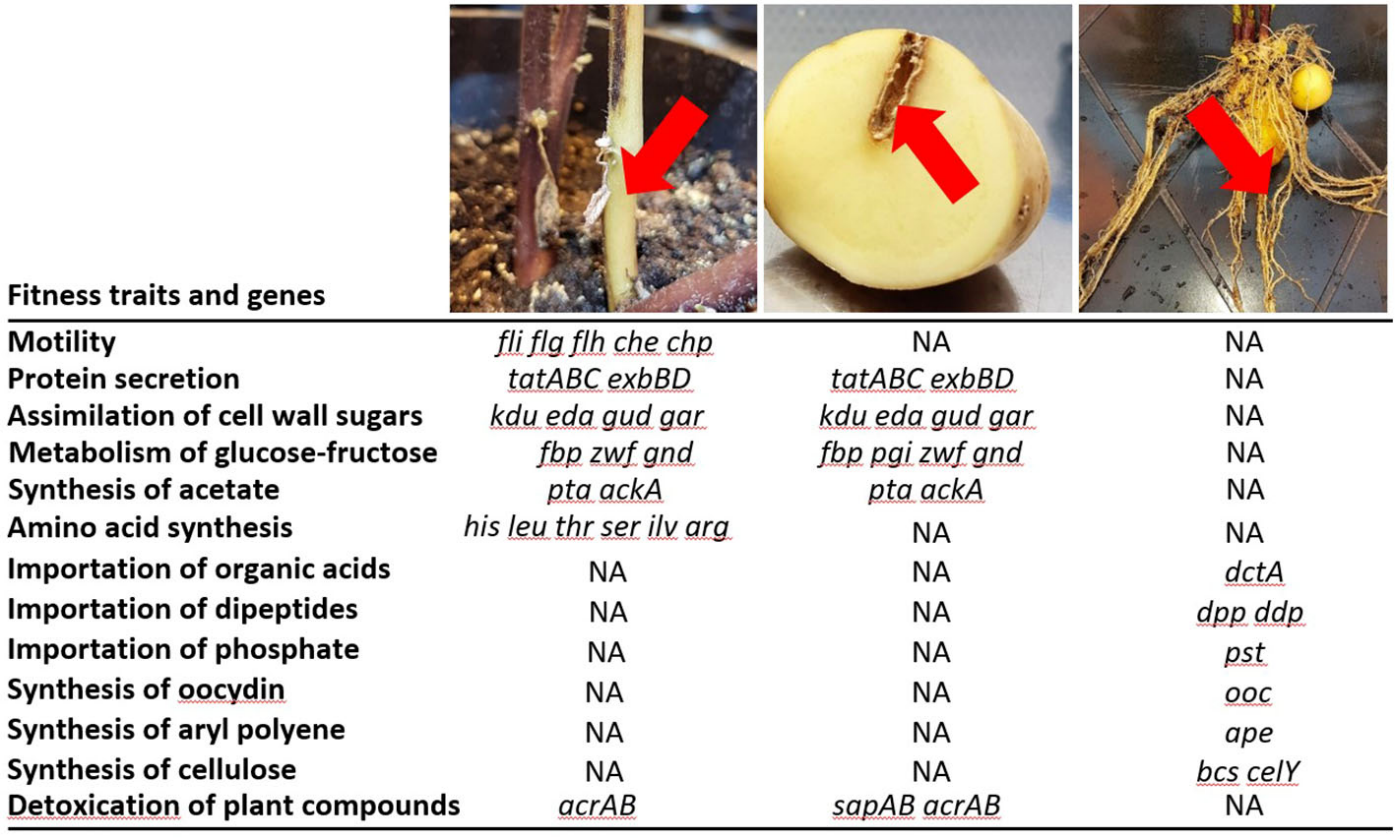
*Dickeya solani* pathways associated with competitive growth on roots and lesions in stems and tubers of *Solanum tuberosum*. From left to right, pictures and arrows indicate the plant tissues that are colonized by *D. solani* and analyzed by Tn-seq. For each condition, some genes and pathways are indicated (complete lists of fitness genes are available in **Table S2**). NA indicates that genes were not associated with a variation of fitness in a given condition according to Tn-seq analyses.

In the root colonization condition, no symptoms on roots were observed: pathogens reached 1.9 × 10^5^ ± 1.6 × 10^5^ CFU/g of roots. Forty root systems were grouped into eight lots that represent eight replicates. Tn-seq analysis revealed 83 genes (**Table S2**), which are clustered in four genomic regions (**Figure 3**). None of them was in common with those identified in the macerated stem and tuber conditions (**Figure 3**, **4**). In the first identified cluster, 11 of 13 adjacent genes were predicted to be involved in the synthesis of oocydin (*oocJKLMNRSTUVW*). In the second cluster, 31 adjacent genes code for C4-dicarboxylic acid transporter (*dctA*), cellulose biosynthesis (*celY-bcsABCDQO*), a putative dipeptide ABC-transporter (*dppABCDF*), conversion of 2-keto-3-deoxy-gluconate (*kdgK*), and some other less characterized functions. In the third cluster (21 adjacent genes), some genes are associated with biosynthesis of an aryl polyene (*apeDEFGHIJKLMNOPQR*) and a phosphate ABC-transporter (*pstABCS*). In the fourth cluster, 18 adjacent genes code for another putative dipeptide ABC-transporter (*ddpABC*), conversion of fructuronate (*yeiQ*), and some other poorly characterized functions. Among the genes pinpointed by Tn-seq in the root condition, two genes (*kdgK* and *yeiQ*) were also important in M9 medium supplemented with glucuronate as a carbon source (**Figure 2**).

### In planta behavior of the Dickeya solani mutants ΔbcsA, ΔddpA, ΔapeH, and ΔpstA

To analyze further some fitness genes associated with root colonization, we constructed in-frame deletion mutants of four genes: *dppA* (=DS0823_v1_2392), encoding an oligopeptide transporter; *pstA* (=DS0823_v1_2527), a phosphate transporter; *bcsA* (=DS0823_v1_2374), involved in cellulose synthesis; and *apeH* (=DS0823_v1_2513), involved in synthesis of an aryl-polyene.

**Figure 5:**
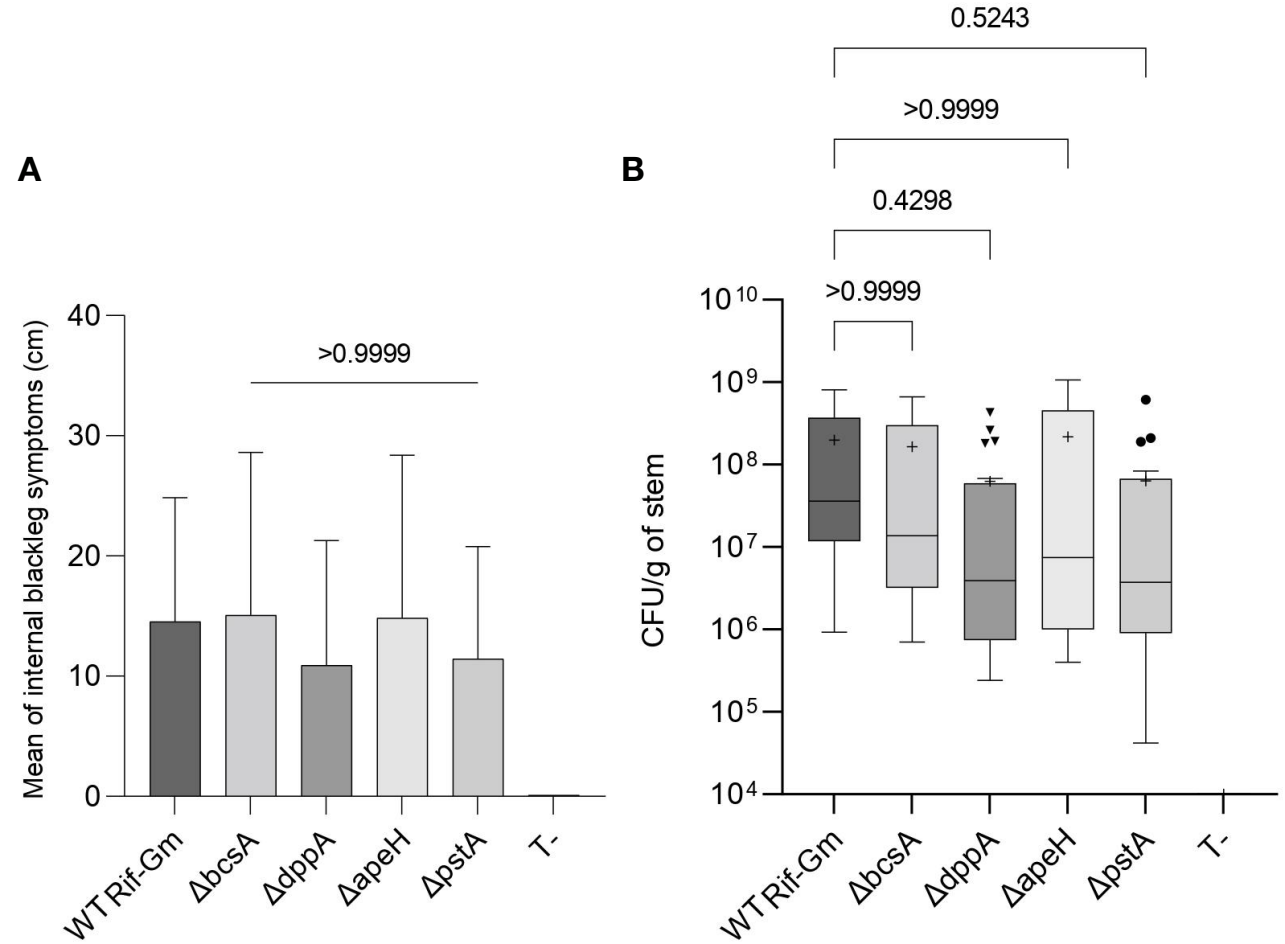
Virulence of the *Dickeya solani* constructed mutants Δ*dppA*, Δ*apeH*, Δ*pstA*, and Δ*bcsA*. Five days after infection in *S. tuberosum* stems, we measured length of lesions in cm **(A)** and enumerated *D. solani* pathogens (CFU/g of macerated tissues) **(B)**. Two independent mutants of each genotype Δ*dppA*, Δ*apeH*, Δ*pstA*, and Δ*bcsA* were analyzed (20 infected stems per genotype) and compared to *D. solani* RNS 08.23.3.1A Rif^R^-Gm^R^ (10 infected stems). The strain *D. solani* RNS 08.23.3.1A Rif^R^-Gm^R^ is indicated as WT Rif-Gm in the graph. Uninoculated stems (T-) did not develop symptoms. In A, Kruskal–Wallis test compared the six conditions (Kruskal–Wallis statistic = 50.52; *p*-value <0.0001; DF = 5) and then Dunn test compared the symptom severity of each mutant genotype with that of *D. solani* RNS 08.23.3.1A Rif^R^-Gm^R^. The *p*-value of the Dunn test is shown: all *D. solani* genotypes caused similar symptoms. In B, center lines show the medians; the crosses show the mean values; box limits are the 25th and 75th percentiles; whiskers extend 1.5 times the interquartile distance; outliers are represented by dots. The statistical analysis was carried out by a Kruskal–Wallis test (Kruskal–Wallis statistic = 54.20; *p*-value <0.0001; DF = 5) followed by a Dunn test to compare each mutant genotype with *D. solani* RNS 08.23.3.1A Rif^R^-Gm^R^. The *p*-values of the Dunn test are indicated on the graph: all *D. solani* genotypes reached a similar abundance in lesions.

We evaluated the capability of the constructed mutants in *D. solani* to provoke blackleg symptoms when injected directly in the stem of the potato plant. The mutants caused similar lesions to those of the wild-type strain (**Figure 5A**). Moreover, mutants and wild-type strains colonized stem lesions at a similar abundance (**Figure 5B**). Thus, a deletion of these genes did not abolish the virulence program of *D. solani* when injected directly in plant tissues.

**Figure 6:**
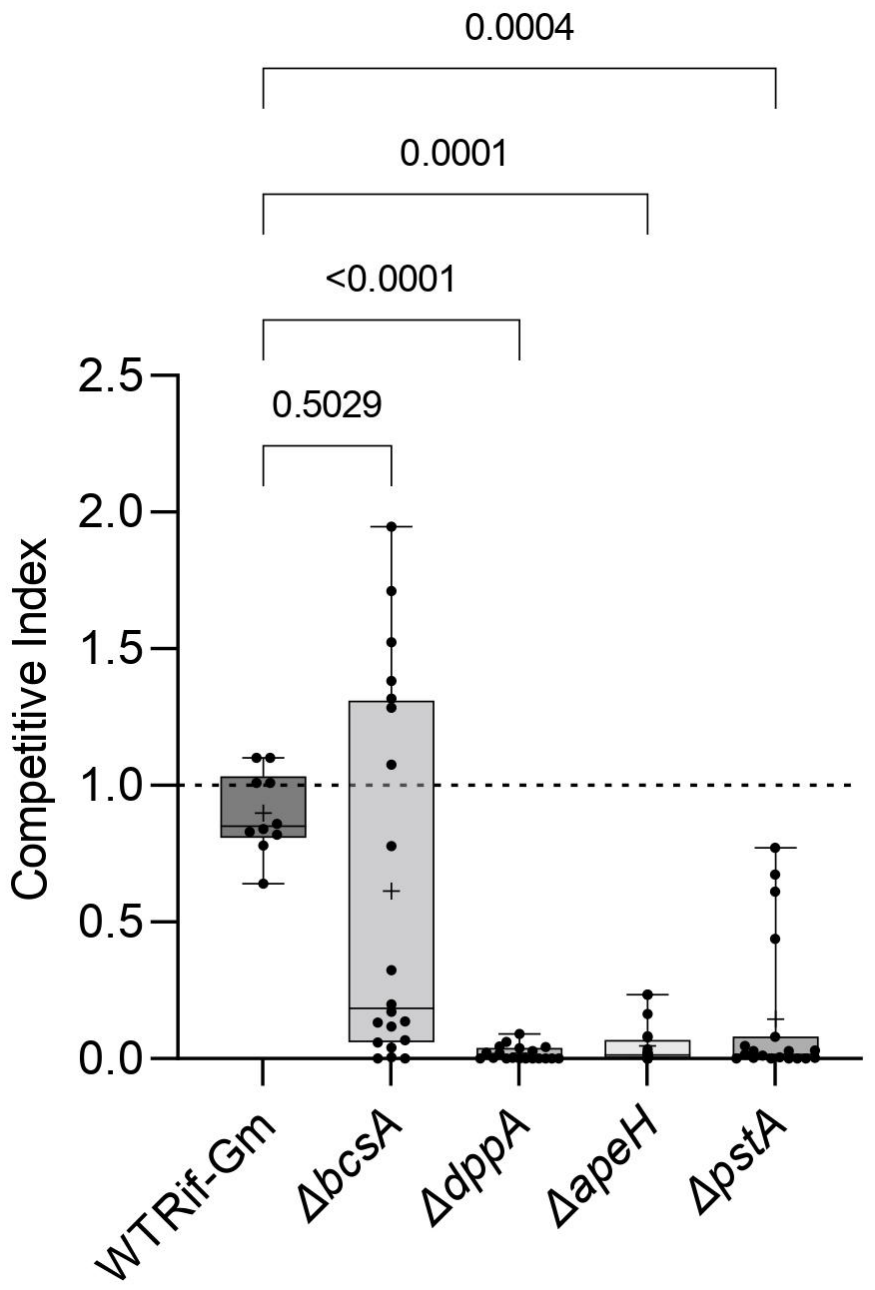
Competitivity of the *D. solani* constructed mutants Δ*dppA*, Δ*apeH*, Δ*pstA*, and Δ*bcsA* in root colonization assays. Two independent clones of each genotype Δ*dppA*, Δ*apeH*, Δ*pstA*, and Δ*bcsA* and *D. solani* RNS 08.23.3.1A Rif^R^-Gm^R^ (indicated as WT Rif-Gm) were challenged with their ancestor *D. solani* RNS 08.23.3.1A Rif^R^ in root colonization assays. Competitive index (CI) values were determined in 10 inoculated plants for each constructed clone (hence 20 per mutant genotype) and 10 plants for the RNS 08.23.3.1A Rif^R^-Gm^R^. Center lines show the medians; crosses show the mean value; box limits indicate the 25th and 75th percentiles as determined by Prism software; whiskers extend from the minimum to maximum values. Statistical analysis was performed with the Kruskal–Wallis test (Kruskal–Wallis statistic = 37.64; *p*-value <0.0001; DF = 4) and then CI values of the mutants were compared with those of RNS 08.23.3.1A Rif^R^-Gm^R^ (*post-hoc* Dunn’s multiple comparison tests).

Then, we evaluated the competitive fitness of these mutants in root colonization when they were challenged by their ancestor (RNS 08.23.3.1A Rif^R^). A control competition between RNS 08.23.3.1A Rif^R^-Gm^R^ and RNS 08.23.3.1A Rif^R^ was performed to evaluate a potential bias caused by the presence of a Gm-resistance cassette (strains are described in **Table 1**). Eight weeks post-inoculation, the pathogens living on roots were enumerated on selective media and a CI value was calculated for each replicate. CI values of the competitions between RNS 08.23.3.1A Rif^R^ and RNS 08.23.3.1A Rif^R^-Gm^R^ did not differ from one (Dunn test, *p*-value >0.9999). By contrast, median CI of the Δ*dppA*, Δ*apeH*, and Δ*pstA* mutants was below one (0.002, 0.01, and 0.01, respectively), revealing a decrease in the competitive fitness of the constructed mutants (**Figure 6**). These CI values significantly differed from CI values of the reference competition (RNS 08.23.3.1A Rif^R^-Gm^R^ versus RNS 08.23.3.1A Rif^R^). In the case of the Δ*bcsA* mutant, the median CI was also below one (0.19), but the heterogeneity of the CI values between replicates compromised statistical significance (*p*-value = 0.503) when CI values were compared to those of the reference competition RNS 08.23.3.1A Rif^R^-Gm^R^ versus RNS 08.23.3.1A Rif^R^ (**Figure 6**). This fitness variability suggests that multiple environmental parameters could influence the behavior of the Δ*bcsA* mutant during root colonization.

**Figure 7:**
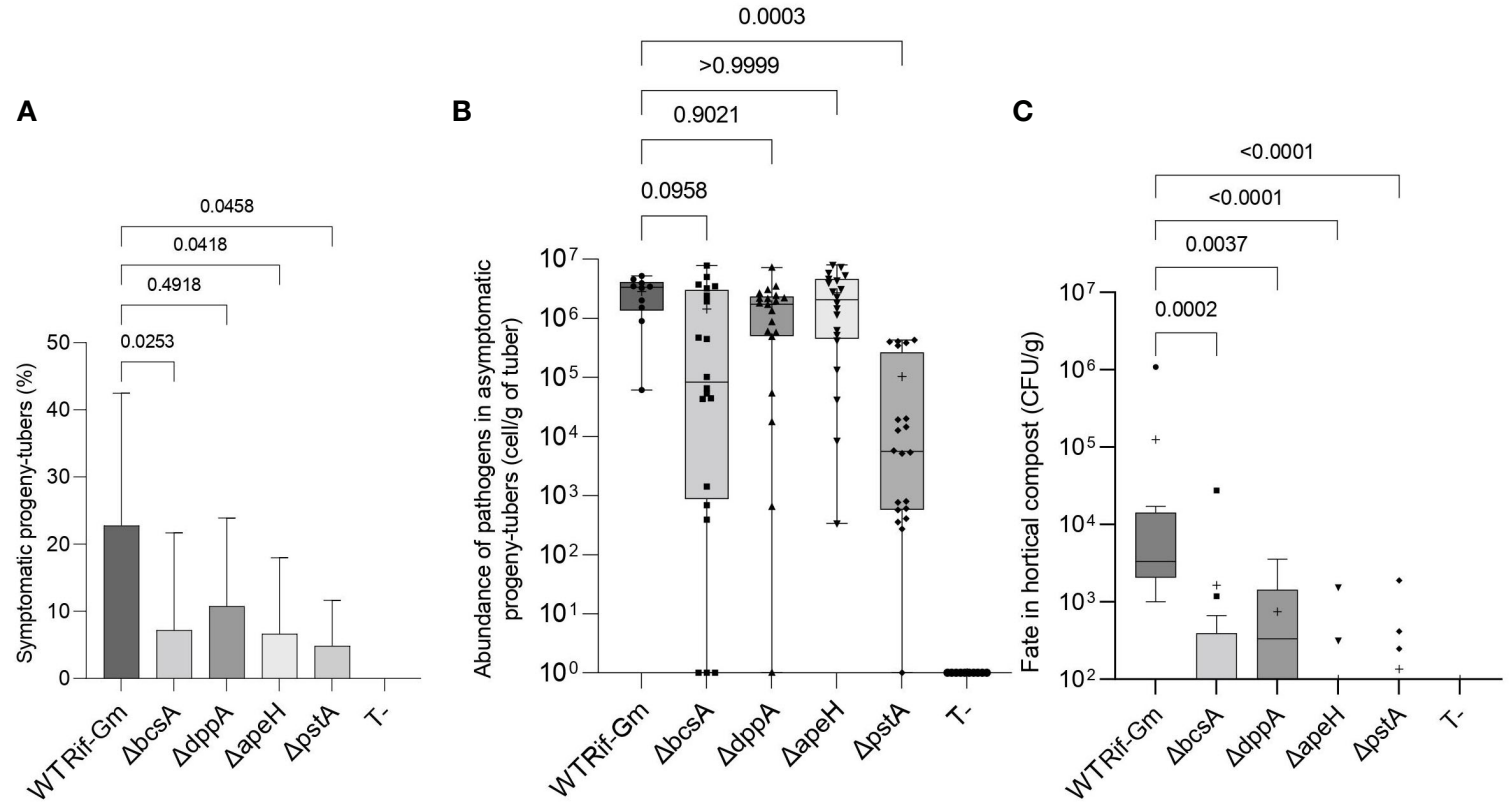
Underground behavior of the *Dickeya solani* constructed mutants Δ*dppA*, Δ*apeH*, Δ*pstA*, and Δ*bcsA*. Two independent mutants of each genotype Δ*dppA*, Δ*apeH*, Δ*pstA*, and Δ*bcsA* and *D. solani* RNS 08.23.3.1A Rif^R^-Gm^R^ (indicated as WT Rif-Gm) were inoculated into potato plant pots by watering. Eight weeks later, progeny tubers were harvested from 20 plants per mutant genotype and 10 plants inoculated by RNS 08.23.3.1A Rif^R^-Gm^R^. The percentage (%) of plants exhibiting blackleg symptoms was determined **(A)** as well as the abundance of pathogens in the asymptomatic progeny tuber **(B)**. In C, pathogens were enumerated in compost in the vicinity of the roots. Statistical analyses were performed with the Kruskal–Wallis test: in A, Kruskal–Wallis statistic = 16.09; *p*-value = 0.0066; DF = 5; in B, Kruskal–Wallis statistic = 48.20; *p*-value <0.0001; DF = 5; in C, Kruskal–Wallis statistic = 48.97; *p*-value <0.0001; DF = 5. The *p*-values of the comparisons between each mutant genotype and RNS 08.23.3.1A Rif^R^-Gm^R^ (*post-hoc* Dunn’s multiple comparison tests) are indicated on the graph.

We further investigated how a deletion in the *bcsA, ddpA, apeH*, and *pstA* genes could modify the underground behavior of *D. solani*. Each pathogen (the four mutants and strain RNS 08.23.3.1A Rif^R^Gm^R^ used as a reference) was inoculated separately by watering the roots with 10^10^ CFU per pot. Eight weeks later, when tuber progeny had developed, we observed that potato plants produced a similar number of progeny tubers (mean value 10.0 tubers ± 0.4) when inoculated by the mutants and RNS 08.23.3.1A Rif^R^Gm^R^ or not inoculated (Kruskal–Wallis test, statistic 7.635, *p*-value= 0.1775). However, the plants inoculated by Δ*bcsA*, Δ*apeH*, and Δ*pstA* mutants exhibited a lower soft-rot disease incidence (% of rotted progeny tubers) when compared to those inoculated by the RNS 08.23.3.1A Rif^R^Gm^R^ strain (Dunn test, *p*-values <0.05; **Figure 7A**). In the same experiment, the Δ*pstA* mutant was significantly impaired in its capacity to colonize asymptomatic progeny tubers as compared to the RNS 08.23.3.1A Rif^R^Gm^R^ strain (Dunn test, *p*-values <0.0005; **Figure 7B**) while the three other mutants did not show a difference compared to the parental strain. Finally, we decided to evaluate the ability of the mutants to survive in the compost at the vicinity of the roots: all four mutants were impaired in this trait, as compared to strain RNS 08.23.3.1A Rif^R^Gm^R^ (**Figure 7C**).

## Discussion

In the pathogen *D. solani*, our Tn-seq analyses highlighted four genomic regions, grouping 13, 18, 21, and 31 adjacent genes, respectively, which may contribute to the pathogen’s capacity to colonize roots of potato plants grown under non-gnotobiotic conditions. These root colonization genes differed from those involved in the competitive proliferation in lesions of tubers (soft-rot disease) and stems (blackleg disease). Hence, this study revealed two contrasting lifestyles of *D. solani* colonizing a potato plant host: an oligotroph lifestyle on roots where it survived at 10^5^ CFU/g of roots, and a copiotroph lifestyle in lesions where *D. solani* proliferation reached 10^9^ to 10^10^ CFU/g of macerated tissues. *D. solani* used different gene repertoires to survive and proliferate under these contrasting conditions: roots and lesions. Because of some limitations in the Tn-seq approach, which we discussed in Torres et al. (2021), we could not exclude the fact that additional genes contribute to an efficient colonization of roots and lesions.

In stem and tuber lesions, carbon metabolism of *D. solani* is oriented towards exploitation of plant cell debris. Comparison of the Tn-seq data acquired *in planta* and in culture media showed that carbon metabolism *in planta* resembled but was not identical to that observed in the presence of pectin and galactarate (**Figure 2**). Notably, *kduD*, involved in the degradation of pectin, was also identified by Tn-seq as a fitness gene in *D. dianthicola* and *D. dadantii* colonizing potato tubers (Helmann et al., 2022). Catabolism of these cell wall derivatives is connected to the upper part of glycolysis/gluconeogenesis (connecting glucose and glyceraldehyde-3P) and to the pentose phosphate pathway (**Figure 2**). In the lower part of glycolysis, the Pta-AckA pathway was also important under maceration conditions (**Table S2**). In *E. coli*, this pathway is involved in ATP production from acetyl-CoA with acetate as a by-product (Schütze et al., 2020). This pathway was also pinpointed in a Tn-seq analysis of *D. dadantii* macerating chicory leaves (Royet et al., 2019). We observed that survival of *D. solani* in lesions is also dependent on its capacity to detoxify plant compounds using the Acr and Sap systems. The Sap transporter is involved in resistance to snakin-1, which is the most abundant antimicrobial peptide in potato tubers (López-Solanilla et al., 1998). In *Erwinia amylovora*, *acr* genes are involved in the resistance to several plant phytoalexins and antibiotics (Al-Karablieh et al., 2009). The Acr efflux pump is also important for the colonization of chicory leaves by *D. dadantii* (Royet et al., 2019). In *D. dadantii*, this efflux system is involved in resistance to a wide spectrum of toxic compounds including the plant antimicrobial peptide thionin (Valecillos et al., 2006). Two important characteristics of the *D. solani* behavior in stem lesions are motility and synthesis of several amino acids (**Figure 4**). Motility may be connected to propagation of the pathogen in plant vessels causing a progressive lesion along the stem. Requirement of amino acid synthesis would reflect an imbalance of carbon and nitrogen resources in cell wall remains. Motility and amino acid synthesis were also highlighted in Tn-seq analyses of *D. dadantii* and *D. dianthicola* proliferating in progressive lesions of chicory leaves and in tubers (Royet et al., 2019; Helmann et al., 2022).

In the root colonization condition, Tn-seq data revealed that *D. solani* coped with another pattern of environmental constraints. Some oligopeptides, organic acids, and mineral phosphate constitute important resources for nutrients that could be imported by the *dpp*, *ddp*, *dctA*, and *pst* transporters (**Figures 3**, **4**). Some sugars (glucoronate and its by-products) would also be assimilated (**Figure 2**). *S. tuberosum* exudates contain a wide variety of molecules, among which glucuronate represents approximately 20% of total sugars (Koroney et al., 2016). *D. solani* reallocated part of its resources to synthesize carbon-rich molecules: cellulose (*celY* and *bcs* genes), antibiotic oocydin A (*ooc* genes), and an aryl polyene (*ape* genes). This suggests a trade-off between proliferation and response to biotic and abiotic stresses. In *D. dadantii*, cellulose synthesis is associated with the formation of biofilm at the air–liquid interface in a carbon-rich culture medium (Mee-Ngan et al., 2005). On roots, the formation of a cellulose biofilm, hence the fitness associated to *bcs* genes, would strongly depend on the abundance of carbon resources and moisture. Variations in these environmental parameters could explain heterogeneity in CI values that we observed when the Δ*bcsA* mutant challenged a wild-type strain (**Figure 6**). In *D. solani* Ds0432.1, *ooc* genes are involved in antibiosis against several Ascomyceta: *Botrytis cinerea, Magnaporthe oryzae*, and *Sclerotinia sclerotiorum* (Brual et al., 2021). Aryl polyenes are present in numerous bacterial genera throughout the Proteobacteria and Bacteroidetes, often in host-associated bacteria (Schöner et al., 2016). Aryl polyenes share a remarkably similar chemical scaffold, consisting of an aryl head group conjugated to a polyene carboxylic acid tail. The biosynthetic pathway is well characterized in several genera, including *Escherichia*, *Vibrio*, and *Xenorhabdus* (Schöner et al., 2016; Johnston et al., 2021). This pigment molecule is exported to the outer membrane. In *E. coli*, it contributes to oxidative stress resistance (H_2_O_2_) and biofilm formation.

The role of *pst* genes is remarkable. In *D. solani*, we observed that a Δ*apeH* mutant was impaired in the competitive colonization of roots and persistence in horticultural compost (**Figures 6**, **7**). In addition, we observed that this locus was important for the colonization of progeny tubers and hence vertical transmission of the pathogens from plant host to its progeny (**Figure 7**). In different pathogens, *pst* genes are involved in phosphate importation as well as in several phosphate-regulated processes including virulence and adherence to surfaces (Daigle et al., 1995; Runyen-Janecky et al., 2005; Röder et al., 2021). In *D. solani* IPO2222, *pstB* was shown to be expressed in the presence of pieces of roots and tubers (Czajkowski et al., 2020), in agreement with an important role in the underground lifestyle of this pathogen.

Overall, this work highlights the versatile lifestyle of *D. solani* through its interaction with the potato host plant: a copiotrophic lifestyle in plant lesions exploits abundant carbon sources and faces plant defense compounds while an oligotrophic lifestyle on roots exploits less abundant, but more diverse carbon resources and competes with microbiota. Several traits and genes related to the underground behavior and proliferation in lesions remain to be investigated further (using complementation of mutants, gene expression monitoring, or structure–function analysis of the encoded proteins) to understand how *D. solani* pathogens efficiently survive on roots, persist in the environment, and colonize progeny tubers.

## Data availability statement

The original contributions presented in the study are publicly available. This data can be found here: NCBI Sequence Read Archives, accession PRJNA939571.

## Author contributions

DF and KR conceived experiments; KR constructed the Tn-library in *D. solani* and prepared the DNA samples that were sequenced by DN at the I2BC platform; KR, GE, and EG constructed the deletion mutants; KR performed inoculation of Tn-library and deletion mutants in tuber, root, and culture medium conditions; KR and EM performed inoculation of Tn-library and deletion mutants in the stem condition. KR performed statistical analyses; KR and DF analyzed the list fitness genes and wrote the manuscript. All authors contributed to the article and approved the submitted version.

## References

[B1] Al-Karablieh N. Weingart H. Ullrich M. S. (2009). The outer membrane protein TolC is required for phytoalexin resistance and virulence of the fire blight pathogen *Erwinia amylovora* . Microb. Biotechnol. 2, 465–475. doi: 10.1111/j.1751-7915.2009.00095.x 21255278 PMC3815907

[B2] Blin P. Robic K. Khayi. S. Cigna J. Munier E. Dewaegeneire P.. (2021). Pattern and causes of the establishment of the invasive bacterial potato pathogen *Dickeya solani* and of the maintenance of the resident pathogen d. dianthicola. Mol. Ecol. 30, 608–624. doi: 10.1111/mec.15751 33226678

[B3] Blin K. Shaw S. Kloosterman A. M. Charlop-Powers Z. Wezel G. P. V. Medema M. H.. (2021). AntiSMASH 6.0: improving cluster detection and comparison capabilities. Nucleic Acids Res. 49, W29–W35. doi: 10.1093/nar/gkab335 33978755 PMC8262755

[B4] Bolger A. M. Lohse M. Usadel B. (2014). Trimmomatic: a flexible trimmer for illumina sequence data. Bioinformatics 30, 2114–2120. doi: 10.1093/bioinformatics/btu170 24695404 PMC4103590

[B5] Bouvier J. T. Sernova N. V. Ghasempur S. Rodionova I. A. Vetting M. W. Al-Obaidi N. F.. (2019). Novel metabolic pathways and regulons for hexuronate utilization in proteobacteria. J. Bacteriol. 201 (2), e00431–e00418. doi: 10.1128/JB.00628-18 PMC6304669 30249705

[B6] Brual T. Effantin G. Baltenneck J. Attaiech L. Groisbois C. Royer M.. (2021). A natural single nucleotide mutation in the small regulatory RNA ArcZ of Dickeya solani switches off the antimicrobial activities against yeast and bacteria. bioRχiv. doi: 10.1101/2021.07.19.452942 PMC10168573 37104544

[B7] Cole B. J. Feltcher M. E. Waters R. J. Wetmore K. M. Mucyn T. S. Ryan E. M.. (2017). Genome-wide identification of bacterial plant colonization genes. PloS Biol. 15, 1–24. doi: 10.1371/journal.pbio.2002860 PMC5627942 28938018

[B8] Czajkowski R. de Boer W. J. Velvis H. van der Wolf J. M. (2010). Systemic colonization of potato plants by a soilborne, green fluorescent protein-tagged strain of dickeya sp. biovar 3. Phytopathology 100, 134–142. doi: 10.1094/PHYTO-100-2-0134 20055647

[B9] Czajkowski R. Fikowicz-Krosko J. Maciag. T. Rabalski L. Czaplewska P. Jafra S.. (2020). Genome-wide identification of *Dickeya solani* transcriptional units up-regulated in response to plant tissues from a crop-host *Solanum tuberosum* and a weed-host *Solanum dulcamara* . Front. Plant Sci. 11. doi: 10.3389/fpls.2020.580330 PMC7492773 32983224

[B10] Daigle F. Fairbrother J. M. Harel J. (1995). Identification of a mutation in the *pst-phoU* operon that reduces pathogenicity of an *Escherichia coli* strain causing septicemia in pigs. Infect. Immun. 63, 4924–4927. doi: 10.1128/iai.63.12.4924-4927 7591158 PMC173707

[B11] Duong D. A. Jensen R. V. Stevens A. M. (2018). Discovery of pantoea stewartii ssp. stewartii genes important for survival in corn xylem through a tn-seq analysis. Mol. Plant Pathol. 19, 1929–1941. doi: 10.1111/mpp.12669 29480976 PMC6638119

[B12] Duprey A. Nasser W. Léonard S. Brochier-Armanet C. Reverchon S. (2016). Transcriptional start site turnover in the evolution of bacterial paralogous genes - the *pelE-pelD* virulence genes in *Dickeya* . FEBS J. 283, 4192–4207. doi: 10.1111/febs.13921 27727510

[B13] Edwards R. A. Keller L. H. Schifferli D. M. (1998). Improved allelic exchange vectors and their use to analyze 987P fimbria gene expression. Gene 207, 149–157. doi: 10.1016/S0378-1119(97)00619-7 9511756

[B14] Galperin M. Y. Makarova K. S. Wolf Y. I. Koonin E. V. (2015). Expanded microbial genome coverage and improved protein family annotation in the COG database. Nucleic Acids Res. 43, D261–D269. doi: 10.1093/nar/gku1223 25428365 PMC4383993

[B15] Golanowska M. Potrykus M. Motyka-Pomagruk A. Kabza M. Bacci G. Galardini M.. (2018). Comparison of highly and weakly virulent *Dickeya solani* strains, with a view on the pangenome and panregulon of this species. Front. Microbiol. 9. doi: 10.3389/fmicb.2018.01940 PMC6127512 30233505

[B16] Gonzalez-Mula A. Lachat J. Mathias L. Naquin D. Lamouche F. Mergaert P.. (2019). The biotroph *Agrobacterium tumefaciens* thrives in tumors by exploiting a wide spectrum of plant host metabolites. New Phytol. 222, 455–467. doi: 10.1111/nph.15598 30447163

[B17] Hélias V. Hamon P. Huchet E. Wolf J. V. D. Andrivon D. (2012). Two new effective semiselective crystal violet pectate media for isolation of *Pectobacterium* and *Dickeya* . Plant Pathol. 61, 339–345. doi: 10.1111/j.1365-3059.2011.02508.x

[B18] Helmann T. C. Deutschbauer A. M. Lindow S. E. (2019). Genome-wide identification of *Pseudomonas syringae* genes required for fitness during colonization of the leaf surface and apoplast. Proc. Natl. Acad. Sci. U.S.A. 116, 18900–18910. doi: 10.1073/pnas.1908858116 31484768 PMC6754560

[B19] Helmann T. C. Filiatrault M. J. Stodghill P. V. (2022). Genome-wide identification of genes important for growth of *Dickeya dadantii* and *Dickeya dianthicola* in potato *(Solanum tuberosum*) tubers. Front. Microbiol. 13. doi: 10.3389/fmicb.2022.778927 PMC8821946 35145503

[B20] Hooven T. A. Catomeris A. J. Akabas L. H. Randis T. M. Maskell D. J. Peters S. E.. (2016). The essential genome of *Streptococcus agalactiae* . BMC Genomics 17, 406. doi: 10.1186/s12864-016-2741-z 27229469 PMC4881062

[B21] Jackson S. A. Fellows B. J. Fineran P. C. (2020). Complete genome sequences of the *Escherichia coli* donor strains ST18 and MFD *pir* . Microbiol. Resour Announc. 9, e01014-20. doi: 10.1128/MRA.01014-20 33154010 PMC7645665

[B22] Johnston I. Osborn L. J. Markley R. L. McManus E. A. Kadam A. Schultz K. B.. (2021). Identification of essential genes for *Escherichia coli* aryl polyene biosynthesis and function in biofilm formation. Biofilms Microbiomes 7, 56. doi: 10.1038/s41522-021-00226-3 34215744 PMC8253772

[B23] Khayi S. Blin P. Chong T. M. Robic K. Chan K. G. Faure D. (2018). Complete genome sequences of the plant pathogens *Dickeya solani* RNS 08.23.3.1.A and *Dickeya dianthicola* RNS04.9. Genome Announcements 6 (4), e01447–e01417. doi: 10.1128/genomeA.01447-17 29371347 PMC5786673

[B24] Khayi S. Blin P. Pédron J. Chong T. M. Chan K. G. Moumni M.. (2015). Population genomics reveals additive and replacing horizontal gene transfers in the emerging pathogen *Dickeya solani* . BMC Genomics 16, 788. doi: 10.1186/s12864-015-1997-z 26467299 PMC4607151

[B25] Koroney A. S. Plasson C. Pawlak B. Sidikou R. Driouich A. Menu-Bouaouiche L.. (2016). Root exudate of *Solanum tuberosum* is enriched in galactose-containing molecules and impacts the growth of *Pectobacterium atrosepticum* . Ann. Bot. 118, 797–808. doi: 10.1093/aob/mcw128 27390353 PMC5055634

[B26] Kuivanen J. Biz A. Richard P. (2019). Microbial hexuronate catabolism in biotechnology. AMB Express. 9 (1), 16. doi: 10.1186/s13568-019-0737-1 30701402 PMC6353982

[B27] Langmead B. Trapnell C. Pop M. Salzberg S. L. (2009). Ultrafast and memory-efficient alignment of short DNA sequences to the human genome. Genome Biol. 10 (3), R25. doi: 10.1186/gb-2009-10-3-r25 19261174 PMC2690996

[B28] Liu Z. Beskrovnaya P. Melnyk R. A. Hossain S. S. Khorasani S. O’Sullivan L. R.. (2018). A genome-wide screen identifies genes in rhizosphere-associated *Pseudomonas* required to evade plant defenses. mBio 9 (6), e00433–e00418. doi: 10.1128/mBio.00433-18 30401768 PMC6222131

[B29] López-Solanilla E. García-Olmedo F. Rodríguez-Palenzuela P. (1998). Inactivation of the *sapA* to *sapF* locus of *Erwinia chrysanthemi* reveals common features in plant and animal bacterial pathogenesis. Plant Cell 10, 917–924. doi: 10.1105/tpc.10.6.917 9634580 PMC144037

[B30] Luneau J. S. Baudin M. Quiroz-Monnens T. Carrère S. Bouchez O. Jardinaud F.. (2022). Genome-wide identification of fitness determinants in the *Xanthomonas campestris* bacterial pathogen during early stages of plant infection. New Phytol. 236, 235–248. doi: 10.1111/nph.18313 35706385 PMC9543026

[B31] Macho A. P. Guidot A. Barberis P. Beuzón C. R. Genin S. (2010). A competitive index assay identifies several *Ralstonia solanacearum* type III effector mutant strains with reduced fitness in host plants. Mol. Plant-Microbe Interact. 23, 1197–1205. doi: 10.1094/MPMI-23-9-1197 20687809

[B32] Matilla M. A. Monson R. E. Murphy A. Schicketanz M. Rawlinson A. Duncan C.. (2022). Solanimycin: biosynthesis and distribution of a new antifungal antibiotic regulated by two quorum-sensing systems. mBio 13 (6), e0247222. doi: 10.1128/mbio.02472-22 36214559 PMC9765074

[B33] Matilla M. A. Stöckmann H. Leeper F. J. Salmond G. P. C. (2012). Bacterial biosynthetic gene clusters encoding the anti-cancer haterumalide class of molecules. J. Biol. Chem. 287, 39125–39138. doi: 10.1074/jbc.M112.401026 23012376 PMC3493953

[B34] Mee-Ngan Y. Yang C. H. Barak J. D. Jahn C. E. Charkowski A. O. (2005). The *Erwinia chrysanthemi* type III secretion system is required for multicellular behavior. J. Bacteriol. 187, 639–648. doi: 10.1128/JB.187.2.639-648 15629935 PMC543537

[B35] Morinière L. Mirabel L. Gueguen E. Bertolla F. (2022). A comprehensive overview of the genes and functions required for lettuce infection by the hemibiotrophic phytopathogen *Xanthomonas hortorum* pv. *vitians* . mSystems 7, e01290–e01221. doi: 10.1128/msystems.01290-21 35311560 PMC9040725

[B36] Olson E. R. Dunyak D. S. Jurss L. M. (1991). Identification and characterization of *dppA*, an *Escherichia coli* gene encoding a periplasmic dipeptide transport protein. J. Bacteriol. 173 (1), 234–244. doi: 10.1128/jb.173.1.234-244.1991 1702779 PMC207180

[B37] Pédron J. Schaerer S. Kellenberger I. Van Gijsegem F. (2021). Early emergence of *Dickeya solani* revealed by analysis of *Dickeya* diversity of potato blackleg and soft rot causing pathogens in switzerland. Microorganisms 9, 1187. doi: 10.3390/microorganisms9061187 34072830 PMC8226965

[B38] Peters J. M. Koo B. M. Patino R. Heussler G. E. Hearne C. C. Qu J.. (2019). Enabling genetic analysis of diverse bacteria with mobile-CRISPRi. Nat. Microbiol. 4, 244–250. doi: 10.1038/s41564-018-0327-z 30617347 PMC6424567

[B39] Potrykus M. Golanowska M. Hugouvieux-Cotte-Pattat N. Lojkowska E. (2015). Regulators involved in *Dickeya solani* virulence, genetic conservation, and functional variability. Mol. Plant Microbe Interact. 1, 57–68. doi: 10.1094/MPMI-99-99-0003-R 27839073

[B40] Potrykus M. Hugouvieux-Cotte-Pattat N. Lojkowska E. (2018). Interplay of classic exp and specific vfm quorum sensing systems on the phenotypic features of *Dickeya solani* strains exhibiting different virulence levels. Mol. Plant Pathol. 19, 1238–1251. doi: 10.1111/mpp.12614 28921772 PMC6638156

[B41] Prigent-Combaret C. Zghidi-Abouzid O. Effantin G. Lejeune P. Reverchon S. Nasser W. (2012). The nucleoid-associated protein Fis directly modulates the synthesis of cellulose, an essential component of pellicle–biofilms in the phytopathogenic bacterium dickeya dadantii. Mol. Microbiol. 86, 172–186. doi: 10.1111/j.1365-2958.2012.08182.x 22925161

[B42] Pritchard J. R. Chao M. C. Abel S. Davis B. M. Baranowski C. Zhang Y. J.. (2014). ARTIST: high-resolution genome-wide assessment of fitness using transposon-insertion sequencing. PloS Genet. 10, e1004782. doi: 10.1371/journal.pgen.1004782 25375795 PMC4222735

[B43] Raoul des Essarts Y. Pédron J. Blin P. Van Dijk E. Faure D. Van Gijsegem F. (2019). Common and distinctive adaptive traits expressed in *Dickeya dianthicola* and *Dickeya solani* pathogens when exploiting potato plant host. Environ. Microbiol. 21, 1004–1018. doi: 10.1111/1462-2920.14519 30618082

[B44] Röder J. Felgner P. Hensel M. (2021). Single-cell analyses reveal phosphate availability as critical factor for nutrition of *Salmonella enterica* within mammalian host cells. Cell. Microbiol. 10, e13374. doi: 10.1111/cmi.13374 34160116

[B45] Royet K. Parisot N. Rodrigue A. Gueguen E. Condemine G. (2019). Identification by tn-seq of *Dickeya dadantii* genes required for survival in chicory plants. Mol. Plant Pathol. 20, 287–306. doi: 10.1111/mpp.12754 30267562 PMC6637903

[B46] Runyen-Janecky L. J. Boyle A. M. Kizzee A. Liefer L. Payne S. M. (2005). Role of the pst system in plaque formation by the intracellular pathogen *Shigella flexneri* . Infect. Immun. 73, 1404–1410. doi: 10.1128/IAI.73.3.1404-1410 15731038 PMC1064976

[B47] Schöner T. A. Gassel S. Osawa A. Tobias N. J. Okuno Y. Sakakibara Y.. (2016). Aryl polyenes, a highly abundant class of bacterial natural products, are functionally related to antioxidative carotenoids. Chem Bio Chem 17, 247–253. doi: 10.1002/cbic.201500474 26629877

[B48] Schütze A. Benndorf D. Püttker S. Kohrs F. Bettenbrock K. (2020). The impact of *ackA*, *pta*, and *ackA*-*pta* mutations on growth, gene expression and protein acetylation in *Escherichia coli* K-12. Front. Microbiol. 11. doi: 10.3389/fmicb.2020.00233 PMC7047895 32153530

[B49] Sivakumar R. Ranjani J. Vishnu U. S. Jayashree S. Lozano G. L. Miles J.. (2019). Evaluation of INSeq to identify genes essential for *Pseudomonas aeruginosa* PGPR2 corn root colonization. G3 (Bethesda) 9, 651–661. doi: 10.1534/g3.118.200928 30705119 PMC6404608

[B50] Su Y. Xu Y. Liang H. Yuan G. Wu X. Zheng D. (2021). Genome-wide identification of *Ralstonia solanacearum* genes required for survival in tomato plants. mSystems 6, e00838–e00821. doi: 10.1128/mSystems.00838-21 34636662 PMC8510521

[B51] Tervo C. J. Reed J. L. (2012). FOCAL: an experimental design tool for systematizing metabolic discoveries and model development. Genome Biol. 13, R116. doi: 10.1186/gb-2012-13-12-r116 23236964 PMC4056367

[B52] Torres M. Jiquel A. Jeanne E. Naquin D. Dessaux Y. Faure D. (2021). *Agrobacterium tumefaciens* Fitness genes involved in the colonization of plant tumors and roots. New Phytol. 233, 905–918. doi: 10.1111/nph.17810 34655498

[B53] Valecillos A. M. Palenzuela P. R. López-Solanilla E. (2006). The role of several multidrug resistance systems in *Erwinia chrysanthemi* pathogensis. Mol. Plant-Microbe Interact. 19, 607–613. doi: 10.1094/MPMI-19-0607 16776294

[B54] van der Wolf J. M. Acuña I. De Boer S. H. Brurberg M. B. Cahill G. Charkowski A. O.. (2021). “Diseases caused by pectobacterium and dickeya species around the world,” in Plant diseases caused by dickeya and pectobacterium species. Eds. Van Gijsegem F. van der Wolf J. M. Toth I. K. (Cham: Springer International Publishing), 215–261. doi: 10.1007/978-3-030-61459-1_7

[B55] van der Wolf J. M. Nijhuis E. H. Kowalewska M. J. Saddler G. S. Parkinson N. Elphinstone J. G.. (2014). *Dickeya solani* Sp. nov., a pectinolytic plant-pathogenic bacterium isolated from potato *(Solanum tuberosum*). Int. J. Syst. Evol. Microbiol. 64, 768–774. doi: 10.1099/ijs.0.052944-0 24225027

[B56] Van Gijsegem F. Hugouvieux-Cotte-Pattat N. Kraepiel Y. Lojkowska E. Moleleki L. N. Gorshkov V.. (2021). “Molecular interactions of pectobacterium and dickeya with plants,” in Plant diseases caused by dickeya and pectobacterium species. Eds. Van Gijsegem F. van der Wolf J. M. Toth I. K. (Cham: Springer International Publishing), 85–147. doi: 10.1007/978-3-030-61459-1_4

[B57] Wiles T. J. Norton J. P. Russell C. W. Dalley B. K. Fischer K. F. Mulvey M. A. (2013). Combining quantitative genetic footprinting and trait enrichment analysis to identify fitness determinants of a bacterial pathogen. PloS Genet. 9 (8), e1003716. doi: 10.1371/journal.pgen.1003716 23990803 PMC3749937

[B58] Zhou J. Zhang H. Wu J. Liu Q. Xi P. Lee J.. (2011). A novel multidomain polyketide synthase is essential for zeamine production and the virulence of *Dickeya zeae* . Mol. Plant-Microbe Interact. 24, 1156–1164. doi: 10.1094/MPMI-04-11-0087 21899437

[B59] Zobel S. Benedetti I. Eisenbach L. De Lorenzo V. Wierckx N. Blank L. M. (2015). Tn7-based device for calibrated heterologous gene expression in pseudomonas putida. ACS Synthetic Biol. 4, 1341–1351. doi: 10.1021/acssynbio.5b00058 26133359

