# Supplementary material for: Dissimilar gene repertoires of Dickeya solani involved in the colonization of lesions and roots of Solanum tuberosum
Source: Front Plant Sci. 2023 May 8;14:1154110. doi: 10.3389/fpls.2023.1154110 (PMC10202176; doi:10.3389/fpls.2023.1154110)
Supplement: Supplementary file 1 [file DataSheet_1.pdf]

Supplementary Material

Dissimilar gene repertoires of *Dickeya solani* involved in colonization of lesions and roots of *Solanum tuberosum*

Kévin Robic, Euphrasie Munier, Géraldine Effantin, Joy Lachat, Delphine Naquin, Erwan Gueguen, Denis Faure*.

*** Correspondence:** Corresponding Author: denis.faure@iébc.paris-saclay.fr

1 Supplementary Data

S1 Appendix. Media for bacterial cultures

Dickeya solani RNS 08.23.3.1.A, whose genome has been fully sequenced and assembled (Khayi et al., 2018) was cultivated at 28°C in Tryptone Yeast (TY) medium (Bacto tryptone 5 g/L; yeast extract 3 g/L). TY medium was supplemented with gentamicin (Gm; 10 µg/ml), kanamycin (Km; 50 µg/ml), rifampicin (Rif; 100 µg/ml), chloramphenicol (Cm; at 25µg/mL) and cycloheximide (200 µg/ml). *D. solani* derivatives were also cultivated in M9 synthetic medium (Na_2HPO_4, $12H_2O$ 17,2 g/L; KH_2PO_4 3 g/L; NaCl 2,5 g/L; NH_4Cl 1 g/L, $MgSO_4$ 2 mM; $CaCl_2$ 100 µM) supplemented with different carbon sources (2 g/L). *Escherichia coli* strains were cultivated in a modified Lysogenic Broth (LBm) medium (yeast extract 5 g/L, bacto tryptone 10 g/L, NaCl 5 g/L) at 37°C. LBm was supplemented with Gm (10 µg/ml) and diaminopimelic acid (300 µg/ml). Media were solidified with 1,5% agar.

S2 Appendix. Construction of the Tn-library in *D. solani* RNS 08.23.3.1.A Rif^R

E. coli MFDpir carrying pSamEC (plasmid donor strain) and *D. solani* RNS 08.23.3.1.A rifampicin-resistant (Rif^R, recipient strain) were cultivated in TY medium supplemented with 100 µg/ml of Rif and in LBm medium supplemented with 300 µg/ml diaminopimelic acid. Both cultures were centrifuged and adjusted to 10 unit of OD_{600nm}. Equivalent volumes of cell suspensions were mixed, centrifuged, and suspended in liquid TY medium with diaminopimelic acid at 300 µg/ml. Five hundred spots of the cell mixture (25 mL in total) were deposited on TY medium agar plates supplemented with 300 µg/ml diaminopimelic acid and incubated overnight at 28°C. The spots were suspended in 0,8% NaCl solution and spread on TY medium agar plates supplemented with rifampicin and kanamycin. Serial dilutions and plating were performed to determine the number of Tn-mutants obtained.

S3 Appendix. Construction of the *D. solani* RNS 08.23.3.1.A Rif^R-Gm^R

This integration was obtained by tri-parental conjugation between the donor strain *E. coli* MFDpir carrying pTn7-M, helper strain *E. coli* MFDpir carrying pMobile-CRISPRi_1 and recipient strain *D. solani* RNS 08.23.3.1A Rif^R carrying a spontaneous mutation in the *rpoB* gene. The Gm resistance cassette, carried by the plasmid pTn7-M (Zobel et al., 2015) of the MFDpir strain, was inserted into the unique attTn7 site of the *D. solani* strain using the plasmid pMobile-CRISPRi_1 (Peters et al., 2019) from the helper strain, encoding the transposition pathway specific to the Tn7 site. Transconjugants were selected on LBm supplemented with Rif and Gm, and verified by PCR.

S4 Appendix. Construction of the deletion mutants in *D. solani* RNS 08.23.3.1.A Rif^R-Gm^R

The suicide plasmid pRE112 (Edwards et al., 1998) of the *E. coli* MFDpir strain, containing the *sacB* gene and 500 bp of DNA upstream and downstream of the gene to be deleted was integrated by conjugation into *D. solani* RNS 08.23.3.1A Rif^R-Gm^R strain. The primers used for amplification of the 500 bp of DNA upstream and downstream of the genes are listed in table S3. The conjugation was carried out by mixing the donor and recipient bacteria (ratio 1:1) in an LBm medium agar plate supplemented with diaminopimelic acid (300 µg / mL). The mixture was incubated at 28°C overnight. The integration of the plasmid into the genome of the recipient strain was then selected on LBm selection medium supplemented with chloramphenicol at 25µg/mL. The second recombination event was selected by plating the Cm-resistant transconjugants on LBm agar without NaCl and supplemented with 5% sucrose and Rif. The deletions were checked by PCR (primers in table S3) and Sanger sequencing.

S5 Appendix Calculation of competitive index in competition assays

Abundance of *D. solani* RNS 08.23.3.1A Rif^R-Gm^R and *D. solani* RNS 08.23.3.1A Rif^R in inoculum and plant tissues was quantified onto TY agar medium supplemented with appropriate antibiotics and then used to calculate the competitive index (CI) values according to the formula (Macho et al., 2010):

$$Competitive\ index = \frac{(\ D.solani\ mutant\ \div D.solani\ total\ )symptoms}{(\ D.solani\ mutant\ \div D.solani\ total\ )inoculum}$$

2 Supplementary Figures and Tables

S1 Table. Genome annotation of the *D. solani* RNS 08.23.3.1A genome (Excel file)

Sheet 1 : MAGE annotation of *D. solani* RNS 08.23.3.1A

Sheet 2 : Correspondence between NCBI and MAGE annotations of *D. solani* RNS 08.23.3.1A

Sheet 3 : List of *D. solani* RNS 08.23.3.1A genes without any TA site

S2 Table. Tn-seq analyses of the *D. solani* Tn-mutant library (Excel file)

Sheet 1 : El-ARTIST analysis of the Tn-mutant library of *D. solani* in TY culture condition

Sheet 2 : El-ARTIST analysis of the Tn-mutant library of D. solani in M9-sucrose culture condition

Sheet 3 : Con-ARTIST analysis of the Tn-mutant library in M9-pectin vs M9-sucrose culture condition

Sheet 4 : Con-ARTIST analysis of the Tn-mutant library in M9-galacturonate vs M9-sucrose condition

Sheet 5 : Con-ARTIST analysis of the Tn-mutant library in M9-glucuronate vs M9-sucrose condition

Sheet 6 : Con-ARTIST analysis of the Tn-mutant library in M9-galactarate vs M9-sucrose condition

Sheet 7 : Con-ARTIST analysis of the Tn-mutant library in macerated stems vs TY culture condition

Sheet 8 : Con-ARTIST analysis of the Tn-mutant library in roots vs TY culture condition

Sheet 9 : Con-ARTIST analysis of the Tn-mutant library in macerated tubers vs TY culture condition

Sheet 10 : COG analyses for all the Tn-seq lists of fitness genes

S3 Table. Primers used for DNA amplification

Name	5'-Sequence	Usage
P5-primer<br>P7-primer	AATGATACGGCGACCACCGAGATCTACACTCTTTCCCTACACGACGCTCTTCCGATCT<br>CAAGCAGAAGACGGCATACGAGATAGACCGGGGACTTATCATCCAACCTGT	Amplification of Tn-borders
L992-D083_2380<br>L993-D083_2380	TGAACTGCATGAATTCCCGGGAGAGCTCCATTGGTCACCACCTGATCG<br>AGGATAATCGATGAAAGCGGACCTGAAGAAGG	Amplification of 500 bp upstream of *bcsA*
L994-D083_2380<br>L995-D083_2380	CAGGTCCGCTTTCATCGATTATCCTGATATTTATATTGATGG<br>CCGATCCCAAGCTTCTTCTAGAGGTACCGCGTCGTTGTCGCTGATACC	Amplification of 500 bp downstream of *bcsA*
L1024-D083_2380<br>L1025-D083_2380	GTATTGAGCGCCGCCAGTTC<br>ATCTGGTGGTGATGATGGCG	Verification of deleted Δ*bcsA* allele
L996-D083_2397<br>L997-D083_2397	TGAACTGCATGAATTCCCGGGAGAGCTCTCCAGCGTGGCTTTGAAACG<br>TGTAATGGCTAAATCAGTAGATTAAGACTATCTATCACGTC	Amplification of 500 bp upstream of *dppA*
L998-D083_2397<br>L999-D083_2397	TTAATCTACTGATTTAGCCATTACATGAACTCCATTG<br>CCGATCCCAAGCTTCTTCTAGAGGTACCGTCTGATGCTGTTTTTGTGGTG	Amplification of 500 bp downstream of *dppA*
L1026-D083_2397<br>L1027-D083_2397	TCATCGCGCAGACGCC<br>CCTATGAGTTCTTGCGTGGTG	Verification of deleted Δ*dppA* allele
L1000-D083_2519<br>L1001-D083_2519	TGAACTGCATGAATTCCCGGGAGAGCTCGCATGCTGATGCTCAGAGG<br>GTATCTGCGGCCAATTATTCATTCCGTCACACTATTCTTCTATCATC	Amplification of 500 bp upstream of *apeH*
L1002-D083_2519<br>L1003-D083_2519	GGAATGAATAATTGGCCGCAGATACAGGAGCTG<br>CCGATCCCAAGCTTCTTCTAGAGGTACCTCATCGCGCCGTGGTTGTAAC	Amplification of 500 bp downstream of *apeH*
L1028-D083_2519<br>L1029-D083_2519	GCTGTTGGCGCTGTTGTTTC<br>CGTCATCGCTGCCGTCATC	Verification of deleted Δ*apeH* allele
L1004-D083_2534<br>L1005-D083_2534	TGAACTGCATGAATTCCCGGGAGAGCTCTGGTCGGCACCCTGTTCTCC<br>ACGGTTAATGTGTAATGCTCGCCATCAGCGTGC	Amplification of 500 bp upstream of *pstA*
L1006-D083_2534<br>L1007-D083_2534	ATGGCGAGCATTACACATTAACCGTCTTAAGAAAACATATTAAC<br>CCGATCCCAAGCTTCTTCTAGAGGTACCTCGTCCATCTCCGCACGCGAC	Amplification of 500 bp downstream of *pstA*
L1030-D083_2534<br>L1031-D083_2534	GGCAACGTGCTTTCCAATATCC<br>TTGGTCAGCGCCCACTGTAC	Verification of deleted Δ*pstA* allele

S4 Table. Accession numbers of Tn-Seq data (.bam files)

Sheet 1 : List of the .bam sequence read archives (SRA) in the bioproject PRJNA939571 at NCBI

S1 Fig. Position of TA sites in the genome of *Dickeya solani* RNS 08.23.3.1A

Complete genome of *D. solani* RNS 08.23.3.1A [1] was analysed for its abundance in TA site. From the outside to the inside, the tracks represent: forward and reverse coding sequences (in blue), MiscRNA (in green), tRNA (in orange), rRNA in (purple) and number of TA site per Kb (in black).

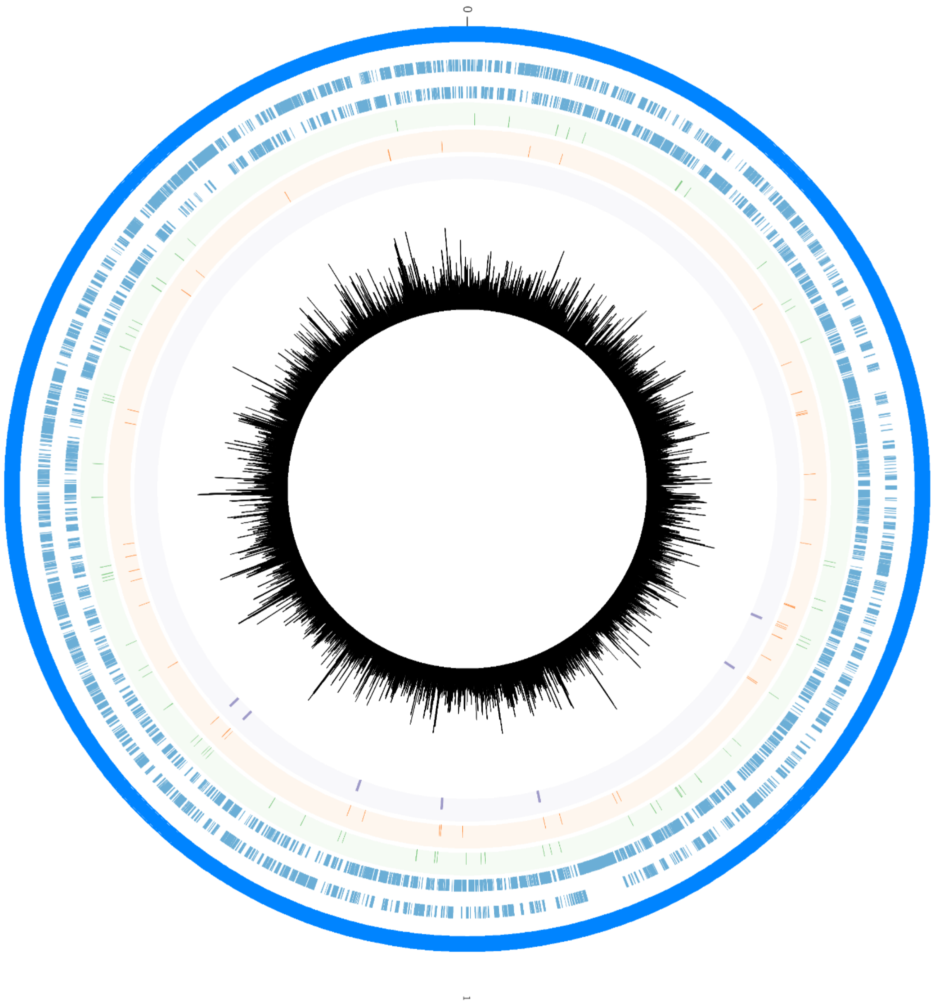

References

Khayi, S., Blin, P., Chong, T.M., Robic, K., Chan, K.G., Faure. D. (2018). Complete genome sequences of the plant pathogens *Dickeya solani* RNS 08.23.3.1.A and *Dickeya dianthicola* RNS04.9. Genome Announcements 6 (4), e01447-17. doi:10.1128/genomeA.01447-17

Zobel, S., Benedetti, I., Eisenbach, L., Lorenzo, V.D., Wierckx, N., Blank, L.M. (2015). Tn7-Based Device for Calibrated Heterologous Gene Expression in *Pseudomonas putida*. ACS Synthetic Biology 4, 1341–1351. doi:10.1021/acssynbio.5b00058

Peters, J.M., Koo, B.M., Patino, R., Heussler, G.E., Hearne, C.C., Qu, J., et al. (2019). Enabling genetic analysis of diverse bacteria with Mobile-CRISPRi. Nature Microbiology. 4, 244–250. doi:10.1038/s41564-018-0327-z

Edwards, R.A., Keller, L.H., Schifferli, D.M. (1998). Improved allelic exchange vectors and their use to analyze 987P fimbria gene expression. Gene 207, 149–157. doi:10.1016/S0378-1119(97)00619-7

Macho, A.P., Guidot, A., Barberis, P., Beuzón, C.R., Genin, S. (2010). A competitive index assay identifies several *Ralstonia solanacearum* type III effector mutant strains with reduced fitness in host plants. Molecular Plant-Microbe Interactions. 23, 1197–1205. doi:10.1094/MPMI-23-9-1197
